# GH18 family glycoside hydrolase Chitinase A of *Salmonella* enhances virulence by facilitating invasion and modulating host immune responses

**DOI:** 10.1371/journal.ppat.1010407

**Published:** 2022-04-28

**Authors:** Kasturi Chandra, Atish Roy Chowdhury, Ritika Chatterjee, Dipshikha Chakravortty

**Affiliations:** 1 Department of Microbiology and Cell Biology, Indian Institute of Science, Bangalore, India; 2 Centre for BioSystems Science and Engineering, Indian Institute of Science, Bangalore, India; University of California Davis School of Medicine, UNITED STATES

## Abstract

*Salmonella* is a facultative intracellular pathogen that has co-evolved with its host and has also developed various strategies to evade the host immune responses. *Salmonella* recruits an array of virulence factors to escape from host defense mechanisms. Previously *chitinase A* (*chiA*) was found to be upregulated in intracellular *Salmonella*. Although studies show that several structurally similar chitinases and chitin-binding proteins (CBP) of many human pathogens have a profound role in various aspects of pathogenesis, like adhesion, virulence, and immune evasion, the role of chitinase in the intravacuolar pathogen *Salmonella* has not yet been elucidated. Therefore, we made chromosomal deletions of the chitinase encoding gene (*chiA*) to study the role of chitinase of *Salmonella enterica* in the pathogenesis of the serovars, Typhimurium, and Typhi using *in vitro* cell culture model and two different *in vivo* hosts. Our data indicate that ChiA removes the terminal sialic acid moiety from the host cell surface, and facilitates the invasion of the pathogen into the epithelial cells. Interestingly we found that the mutant bacteria also quit the *Salmonella*-containing vacuole and hyper-proliferate in the cytoplasm of the epithelial cells. Further, we found that ChiA aids in reactive nitrogen species (RNS) and reactive oxygen species (ROS) production in the phagocytes, leading to MHCII downregulation followed by suppression of antigen presentation and antibacterial responses. Notably, in the murine host, the mutant shows compromised virulence, leading to immune activation and pathogen clearance. In continuation of the study in *C*. *elegans*, *Salmonella* Typhi ChiA was found to facilitate bacterial attachment to the intestinal epithelium, intestinal colonization, and persistence by downregulating antimicrobial peptides. This study provides new insights on chitinase as an important and novel virulence determinant that helps in immune evasion and increased pathogenesis of *Salmonella*.

## Author summary

Chitinases and chitin-binding proteins have been implicated in the pathogenesis of several human pathogens associated with the mucosal barrier. Interestingly, chitinases from the major enteric pathogen, *Salmonella enterica*, were reported to be upregulated during macrophage and epithelial cell infection. Although *Salmonella* Chitinase ChiA (encoded by STM14_0022) shares sequence similarity with the pathogenic chitinases, its role as a virulence determinant remained obscured. Here we aim to investigate the role of chitinase in the context of *Salmonella* pathogenesis using cell culture, mouse, and nematode models. We found that *Salmonella* requires ChiA to remodel the intestinal epithelium and access the host system. In the phagocytes, chitinase-mediated upregulation of nitric oxide (NO) leads to inhibition of MHC-I bound antigen presentation and CD8^+^ T cell proliferation. Furthermore, the absence of ChiA impairs bacterial adhesion and colonization *in vivo*. During the systemic phase in the murine host, *Salmonella* Typhimurium chitinase prevents immune activation and antimicrobial responses. Additionally, in the *Caenorhabditis elegans*, *Salmonella* Typhi chitinase promotes bacterial attachment to the intestinal epithelium and enhances pathogen colonization and persistence in the intestine by downregulating the antimicrobial peptides SPP1 and ABF2. In conclusion, our study provides novel insights into the role of *Salmonella* chitinase as a novel virulence factor.

## Introduction

*Salmonella* is one of the major foodborne pathogens that cause enteric diseases in humans and other mammals. Although *Salmonella*-mediated enteric illnesses can be treated, the high occurrences of drug-resistant strains challenge pathogen eradication. The human gastrointestinal tract is covered with two distinct types of glycan layers- mucin and complex oligosaccharides (glycocalyx) that protect the enterocytes from the environment [1]. An enteric pathogen, like *Salmonella*, should be able to cleave the mucinous layer to gain access to the enterocytes. In various human pathogens, glycoside hydrolases such as sialidases, muraminidases, glucosaminidases, pullulanases, N-acetylgalactosaminidases (GalNAcases), etc. are known to facilitate the bacterial attachment to the host cells [2]. GH18 family protein chitinases and chitin-binding proteins were also found to be involved in the pathogenesis of several human enteric (*Vibrio cholerae*, *Listeria monocytogenes*, *Serratia marcescens*) [3–7] and non-enteric pathogens (*Pseudomonas aeruginosa*, *Legionella pneumophila*) [8–10]. During the infections caused by these pathogens, the commonality of a mucin-rich host-pathogen interface hinted towards a potentially significant role of chitinases and chitin-binding proteins in breaching the mucosal barrier. Furthermore STM0018 encoded chitinase from *Salmonella* Typhimurium strain SL1344 has been implicated in cleaving β1–6 linked LacdiNAc molecules (prevalent on mammalian glycome and invertebrate glycans) along with other chitin-like chains containing β1–4 linkages [11,12]. However, chitinase from *Salmonella* Typhimurium str. LT2 did not have any effect on pathogenesis [13]. *Salmonella* causes infection in the gut mucosal region, which also has a protective mucinous layer. A BLAST search revealed that *Salmonella* Typhimurium exochitinase ChiA (encoded by STM14_0022) showed 20–40% identity with the abovementioned pathogenic proteins. Further, *Salmonella* Typhi chitinase (ChiA; STY0018) is 98% similar to the *S*. Typhimurium SL1344 *chiA* (STM0018) that was reported to be upregulated in the infected macrophages and epithelial cells [14–16].

We infected epithelial cells and phagocytes with the Δ*chiA* mutant strains, and interestingly, we found that the strains lacking *chiA* were invasion defective in epithelial cells. *Salmonella* is known to remodel the host cell surface glycans to facilitate invasion in the epithelial cells [17–19]. We checked the host cell surface glycan modification by lectin-binding assay. Our data suggest that chitinase aids in glycan remodeling by cleaving the terminal sialic acid (Neu5Ac) and Gal-β1,4-GalNAc, thus making the mannose residues accessible to the bacteria for binding. Further, we found that the phagocytes infected with the mutant bacteria produced less antibacterial molecules. Interestingly, the mutants were significantly less virulent, less persistent, and were unable to dampen host antibacterial and immune responses *in vivo*. Moreover, in this study, we demonstrated a novel role of ChiA in facilitating extra-intestinal colonization of *Salmonella* Typhi in *C*. *elegans*. Together our data indicate that chitinase A plays a multifaceted role in *Salmonella* pathogenesis ranging from aiding bacterial invasion in the epithelial cells, enhancing antibacterial NO production by the phagocytes *ex vivo*, to increasing bacterial persistence in the nematodes and regulating cellular and humoral immune responses *in vivo*.

## Results

### Chitinase deletion impairs bacterial invasion in human epithelial cells

**Fig 1:**
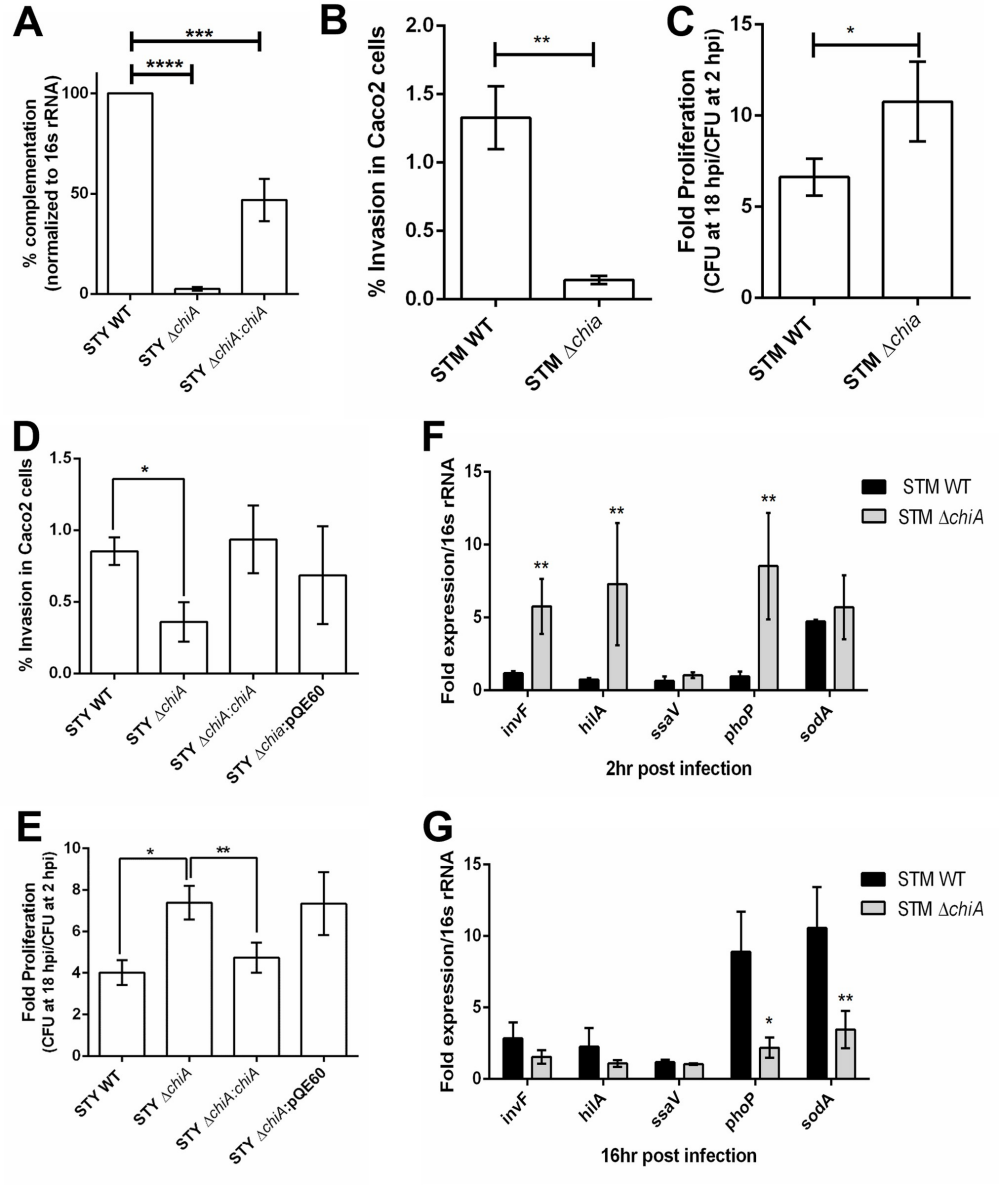
Chitinase deletion impairs bacterial invasion in human epithelial cells. **(A)** *chiA* expression from STY WT, STY Δ*chiA*, STY Δ*chiA*:*chiA* isolated from infected Caco-2 cells 16 hpi. **(B)** % invasion, **(C)** intracellular proliferation of STM WT and STM Δ*chiA* strains, **(D)** % invasion and **(E)** Intracellular proliferation of STY WT, STY Δ*chiA*, STY Δ*chiA*:*chiA* and STY Δ*chiA*:pQE60 strains in Caco-2 cells by gentamicin protection assay. (N = 3, n = 3). Unpaired Student’s t test was used to analyze the data for **B-C** and one-way ANOVA was used to analyze the data for **D-E**. RNA expression level of SPI1 and SPI2 genes in from STM WT and STM Δ*chiA* during **(F)** early phase (2 hpi) and **(G)** late phase (16 hpi) of infection in Caco-2 cells. (N = 3, n = 3); Two-way ANOVA was used to analyze the data.

Since STM ChiA and STY ChiA were 19–24% identical with previously reported pathogenic chitinases and chitin-binding proteins (CBPs) [3–10] (**S1A Fig**), we made isogenic mutants of *chiA* using the lambda red recombinase method (**Tables 1 and 2**) [20]. The trans-complemented strain (STY Δ*chiA*:*chiA*) showed ~50% complementation of *chiA* expression (**Fig 1A)**. The mutants and the complemented strain did not show any growth difference *in vitro* (**S1B and S1C Fig**), suggesting that Chitinase A is non-essential for the extracellular life of *Salmonella sp*. We checked bacterial invasion and intracellular proliferation in Caco-2 cells and found that the *chiA* deletion rendered the bacteria less invasive and hyperproliferative in epithelial cells (**Figs 1B–1E and S1D and S1E**). Inside the host, *Salmonella* SPI1-T3SS effectors induce membrane ruffling that facilitates bacterial entry into the epithelial cells, whereas SPI2 effectors are required for intracellular survival and proliferation [21]. SPI1 effectors *invF* and SPI1 master regulator *hilA* were significantly upregulated in STM Δ*chiA* mutant 2 hours post-infection (hpi), whereas SPI2 encoded *ssaV* expression was similar in both STM WT and STM Δ*chiA* strains at 16 hpi (**Fig 1F and 1G**), suggesting that the reduced bacterial invasion in the epithelial cells by Δ*chiA* mutant is independent of SPI1 gene expression. However, at 16hpi, we also observed a reduction in the expression of *sodA* that encodes superoxide dismutase. SodA protects from oxygen-dependent microbicidal activity [22]; therefore, reduced SodA expression indicates reduced oxidative burst in the infected cells.

**Table 1:** List of strains used in this study.

Strain name	Description	Reference
*S*. Typhimurium ATCC 14028S (STM WT)	Wildtype (WT)	Kind gift from Prof. M. Hensel (Division of Microbiology, University of Osnabr**ü**ck, Germany)
STM Δ*chiA*	Isogenic knockout strain for the gene *chiA*; Kan	This study
STM Δ*invC*	Isogenic knockout strain for the gene *invC*;	Kind gift from Prof. M. Hensel (Division of Microbiology, University of Osnabr**ü**ck, Germany)
*S*. Typhi CT18 (STY WT)	Wildtype (WT)	PGIMER, Chandigarh
STY Δ*chiA*	Isogenic knockout strain for the gene *chiA*; Kan^r^	This study
STY Δ*chiA*:*chiA*	Isogenic complement strain for Δ*chiA* expressing *chiA* under the T5 promoter present in the pQE60 plasmid; Kan^r^ Amp^r^	This study
STY Δ*chiA*:pQE60	Isogenic complement strain with empty pQE60 plasmid; Kan^r^ Amp^r^	This study
mCherry tagged strains	Respective strains carrying pFPV-mCherry plasmid, Amp^r^	This study

**Table 2:** List of primers used in this study.

Gene	Sequence (5’-3’)
STM *chiA* KO FP	TTATGGACCCCGCAGAACGAGCTGCGACAATTTTGAAACGTAAAAGGAAATTTGAAAGTGTAGGCTGGAGCTGCTTC
STM *chiA* KO RP	GGTAAACCAGGGCTTGAATCATGAAGCCCAATACATCGGCTTAATACCGTGTACATATGAATATCCTCCTTAG
STM *chiA* conf FP	GCTGCGACAATTTTGAAAC
STM *chiA* conf RP	GAAGCCCAATACATCGG
STY *chiA* KO FP	GGACCCCGCAGAACGAGCTGCGACAATTTTGAAACGTAAAAGGAAATTTGAAAGTGTAGGCTGGAGCTGCTTC
STY *chiA* KO RP	CCCCGGTAAACCGGGGCTTGAATCATGAAGCCCAATACATCGGCTTAATACCGTGTACATATGAATATCCTCCTTAG
STY *chiA* conf FP	CTGCGACAATTTTGAAACG
STY *chiA* conf RP	CCAATACATCGGCTTAATACC
STY *chiA*:pQE60-*chiA* FP	TACGCCATGGATGGCTACAAGCAAACTGATTCAAG
STY *chiA*:pQE60-*chiA R*P	AGTCGGATCCTTAGTAAGCGCCAAGATCGG
STM/STY *chiA* RT FP	CGGAAGAGGAAGAAGAGATT
STM/STY *chiA* RT RP	CATAGACCACCATTTCACCT
*invF* FP	AGATCGTAAACGCTGCGAGT
*invF* RP	CTGCTGCACAAACGACGAAA
*hilA* FP	GCCGGTGACCATTACGAAGA
*hilA* RP	AAGAGAGAAGCGGGTTGGTG
*ssaV* FP	TATTGATAGGCGCGGACGCTA
*ssaV* RP	CGCCTTATGGGCCATGTCTTT
*phoP* FP	GATCTCTCACGCCGGGAATT
*phoP* RP	TGACATCGTGCGGATACTGG
*sodA* FP	CCTGCCGGTTGAAGAACTGA
*sodA* RP	GGTTGCTGCTGCTTTTTCGA
STM 16s rRNA FP	GTGAGGTAACGGCTCACCAA
STM 16s rRNA RP	TAACCGCAACACCTTCCTCC
*C*. *elegans act2* FP	ATCGTCCTCGACTCTGGAGAT
*C*. *elegans act2* RP	TCACGTCCAGCCAAGTCAAG
*C*. *elegans pmk1* FP	CCAAAAATGACTCGCCGTGA
*C*. *elegans pmk1* RP	CTTTTGCAGTTGGACGACGA
*C*. *elegans mek1* FP	AGCAGCCAATTCCAGAGAGA
*C*. *elegans mek1* RP	CGATCAGTCTGCCAGCAATA
*C*. *elegans clec85* FP	CCAATGGGATGACGGAACCA
*C*. *elegans clec85* RP	CTTCTGTCCAGCCAACGTCT
*C*. *elegans lys7* FP	GTACAGCGGTGGAGTCACTG
*C*. *elegans lys7* RP	GCCTTGAGCACATTTCCAGC
*C*. *elegans ilys2* FP	TGTTGGATCGCTTTCTTGTG
*C*. *elegans ilys2* RP	CATTATGGTTCGGGCCATC
*C*. *elegans spp1* FP	TGGACTATGCTGTTGCCGTT
*C*. *elegans spp1* RP	ACGCCTTGTCTGGAGAATCC
*C*. *elegans abf2* FP	CCGTTCCCTTTTCCTTGCAC
*C*. *elegans abf2* RP	GACGACCGCTTCGTTTCTTG

### Chitinase A facilitates bacterial entry into the epithelial cells by cell surface glycan modification

**Fig 2:**
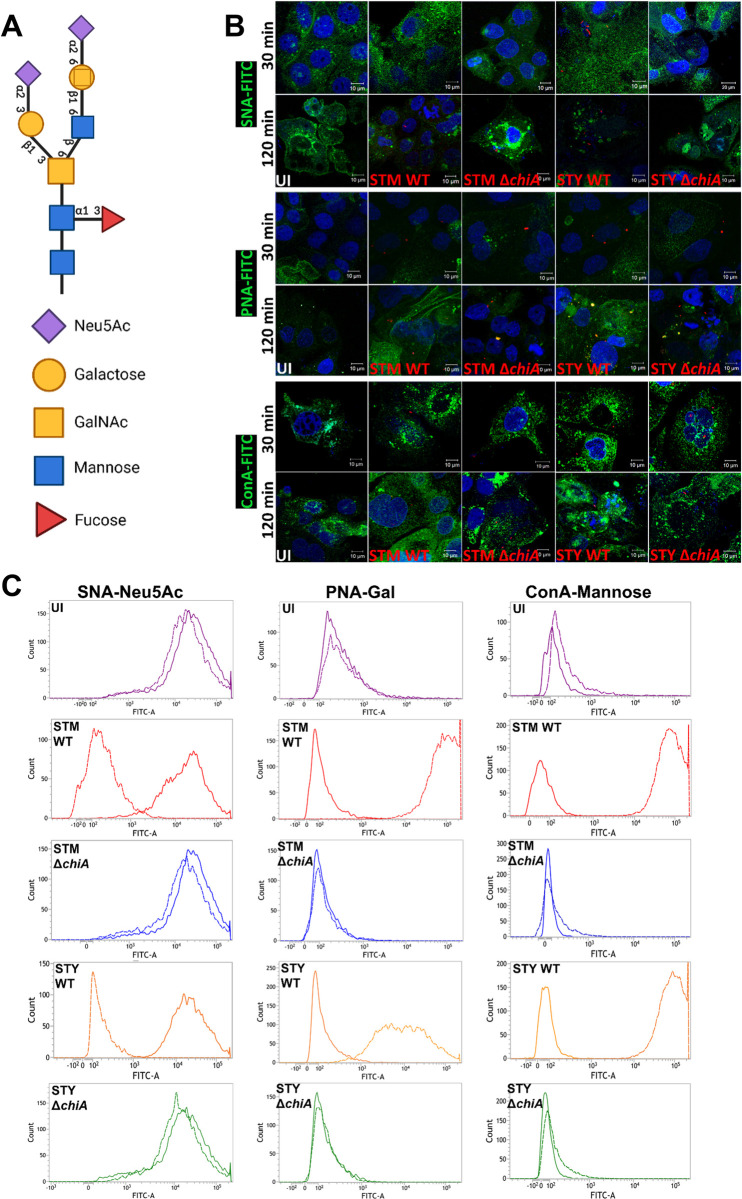
Chitinase aids in glycan remodeling in host epithelial cells. **(A)** Cell surface glycan assembly. **(B)** Representative confocal images of Caco-2 cells stained with SNA-FITC (top panel), PNA-FITC (middle panel) and ConA-FITC (bottom panel) lectin after indicated time intervals of STM WT, STM Δ*chiA*, STY WT and STY Δ*chiA* infection (UI- Uninfected). **(C)** Representative flow cytometry histogram showing the cell surface Neu5Ac-bound SNA-FITC (first column), Gal bound PNA-FITC (second column) and mannose bound ConA-FITC (third column) lectin (UI- Uninfected). Solid lines represent MFI 30 mpi and dashed lines represent MFI 120 mpi. (N = 2).

The intestinal epithelial cells are layered with α2–6, α2–3, α1–3, β1–3 or β1–6 linked glycans on the host epithelial cells in a particular array to form the glycocalyx (**Fig 2A**) [18]. Interestingly, the typhoid toxin from *S*. Typhi also binds to the terminal Neu5Ac (N-Acetylneuraminic acid) moieties to initiate the bacterial attachment to the intestinal epithelium [23]. Therefore, we quantified various glycosyl molecules present on Caco-2 cells after *Salmonella* infection by lectin binding assay. Interestingly, 120 min post-infection (mpi), wildtype (WT) bacteria-infected host cell surface showed a significantly decreased sialylation, but not the Δ*chiA* mutants infected cells, suggesting chitinase is involved in the removal of the terminal sialic acids (**Fig 2B; top panel**). Subsequently, we observed a significant increase in the detection of Gal-β1,3-GalNAc on the WT infected cells compared to the Δ*chiA* infected cells (**Fig 2B; middle panel**). Finally, we observed a substantial increase in the mannose-bound concanavalin A-FITC fluorescence on the surface of WT bacteria-infected cells as compared to the Δ*chiA* infected cells (**Fig 2B; bottom panel**). The cell surface glycan-bound lectin fluorescence was further quantified (**S1F Fig**) and validated by corresponding shift of the glycan-bound FITC lectins using flow cytometry (**Fig 2C)**. Together these data suggested that *Salmonella* chitinase facilitates host cell surface remodeling.

### *Salmonella* ChiA is required for stabilization of the SCVs in epithelial cells

**Fig 3:**
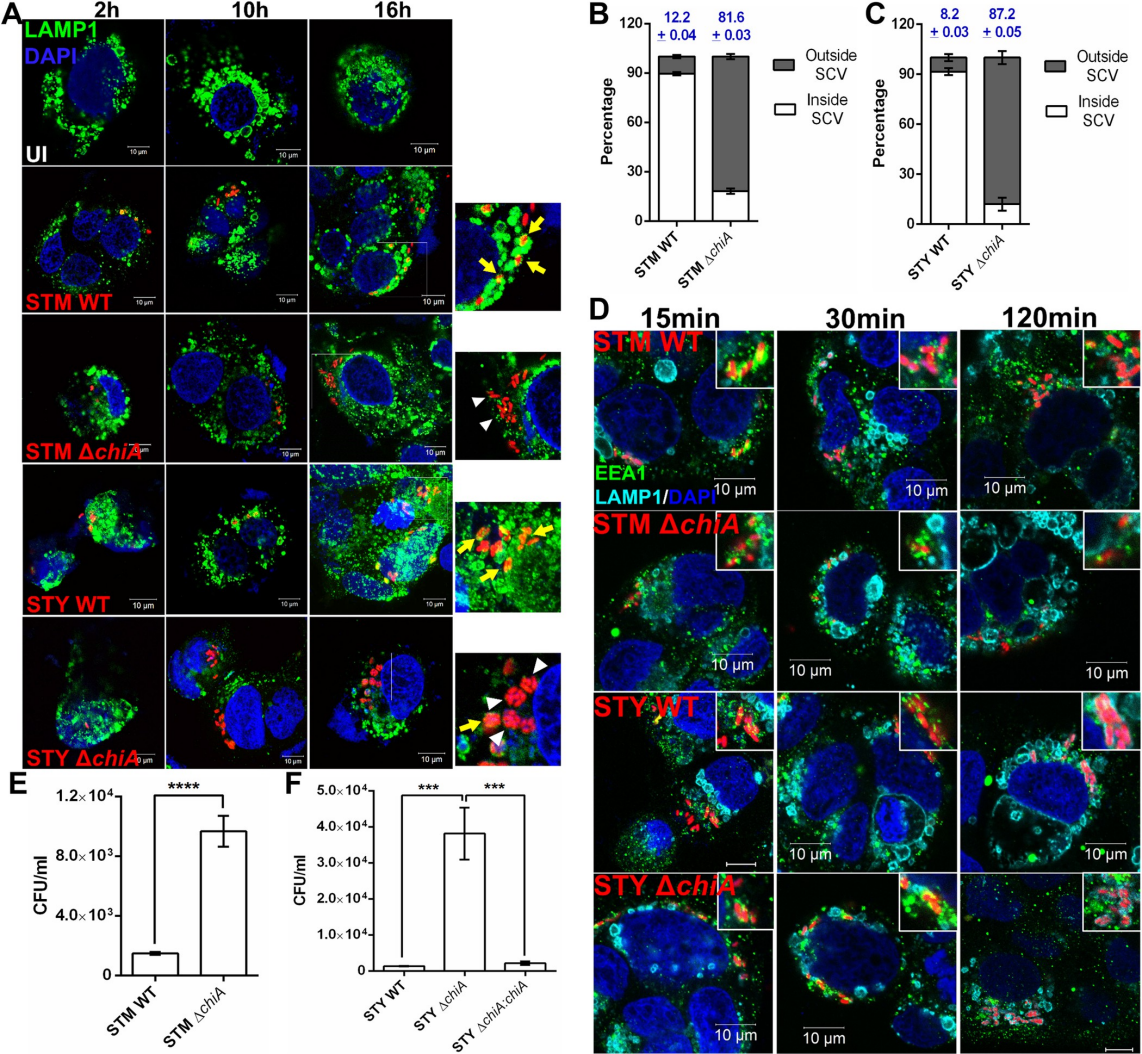
Δ*chiA* mutants quit SCVs in the epithelial cells and hyper-proliferates in the cytoplasm. **(A)** Representative image of Caco-2 cells infected with STM WT, STM Δ*chiA*, STY WT and STY Δ*chiA* strains to visualize the intracellular niche of the bacteria. The SCVs were stained for LAMP1 (UI- Uninfected; Yellow arrows- LAMP1^+^ SCVs, white arrowheads- LAMP1^-^ SCVs). **(B)** % of STM WT and STM Δ*chiA*, **(C)** % of STY WT and STY Δ*chiA* bacteria inside and outside the LAMP1^+^ SCVs 16 hpi was calculated. (N = 3). **(D)** Representative image of Caco-2 cells infected with STM WT, STM Δ*chiA*, STY WT and STY Δ*chiA* strains at MOI 50 to visualize EEA1 and LAMP1 recruitment on the SCVs (scale bars- 10μm; insets show SCVs). Absolute CFU/ml values of **(E)** STM WT and STM Δ*chiA*, and **(F)** STY WT, STY Δ*chiA* and STY Δ*chiA*:*chiA* in Caco-2 cells in chloroquine resistance assay 16 hpi. (N = 3, n = 3). One-way ANOVA was used to analyze the data.

Since several reports suggested that disruption of *S**almonella*-containing vacuoles (SCVs) leads to bacterial hyperproliferation in the cytoplasm of the epithelial cells [24], we checked the intracellular niche of the bacteria in the infected Caco-2 cells. Early SCVs contain early endosomal markers, such as EEA1, Rab4, Rab5, and transferrin receptors, etc., while the late maturation phase is marked by late endosomal markers LAMP1/2, Rab7, Rab11, and vATPases [25]. Interestingly, Δ*chiA* mutant bacteria did not colocalize with the late-endosomal marker LAMP1 at 16 hpi (**Figs 3A** and **S1G and S1H**), suggesting disruption of SCVs in the Δ*chiA* mutant bacteria-infected cells. Upon counting the number of SCV-bound and cytoplasmic bacterial population, we found that 81.6±0.03% of STM Δ*chiA* and 87.2±0.05% of STY Δ*chiA* quit the vacuolar niche compared to the WT bacteria (STM WT 12.2±0.04%, STY WT 8.2±0.03%; **Fig 3B and 3C**). We also found that EEA1, an early endosomal marker, remained associated with the SCVs in WT and Δ*chiA* mutant infected Caco-2 cells at 15–120 mpi, while LAMP1 did not colocalize with the bacteria at early time points (15–30 mpi; **Figs 3D and S1I**). It is known that the cytoplasmic bacteria that escape xenophagy, can hyper-replicate in the cytosol of the epithelial cells [26]. Therefore, we enumerated the cytosolic population by chloroquine (CHQ) resistance assay. Upon transportation into the SCVs by proton pumps present on the SCV membrane, CHQ increases the vacuolar pH and kills the vacuolar *Salmonella*, while the cytosolic bacteria remain viable [27]. Notably, we found a significantly higher number of cytosolic mutant bacteria at 16 hpi (**Fig 3E and 3F**), suggesting that chitinase deletion leads to SCV disruption in epithelial cells and hyper-proliferation in the cytoplasm.

### Chitinase aids in bacterial survival in phagocytes by suppressing antimicrobial responses

**Fig 4:**
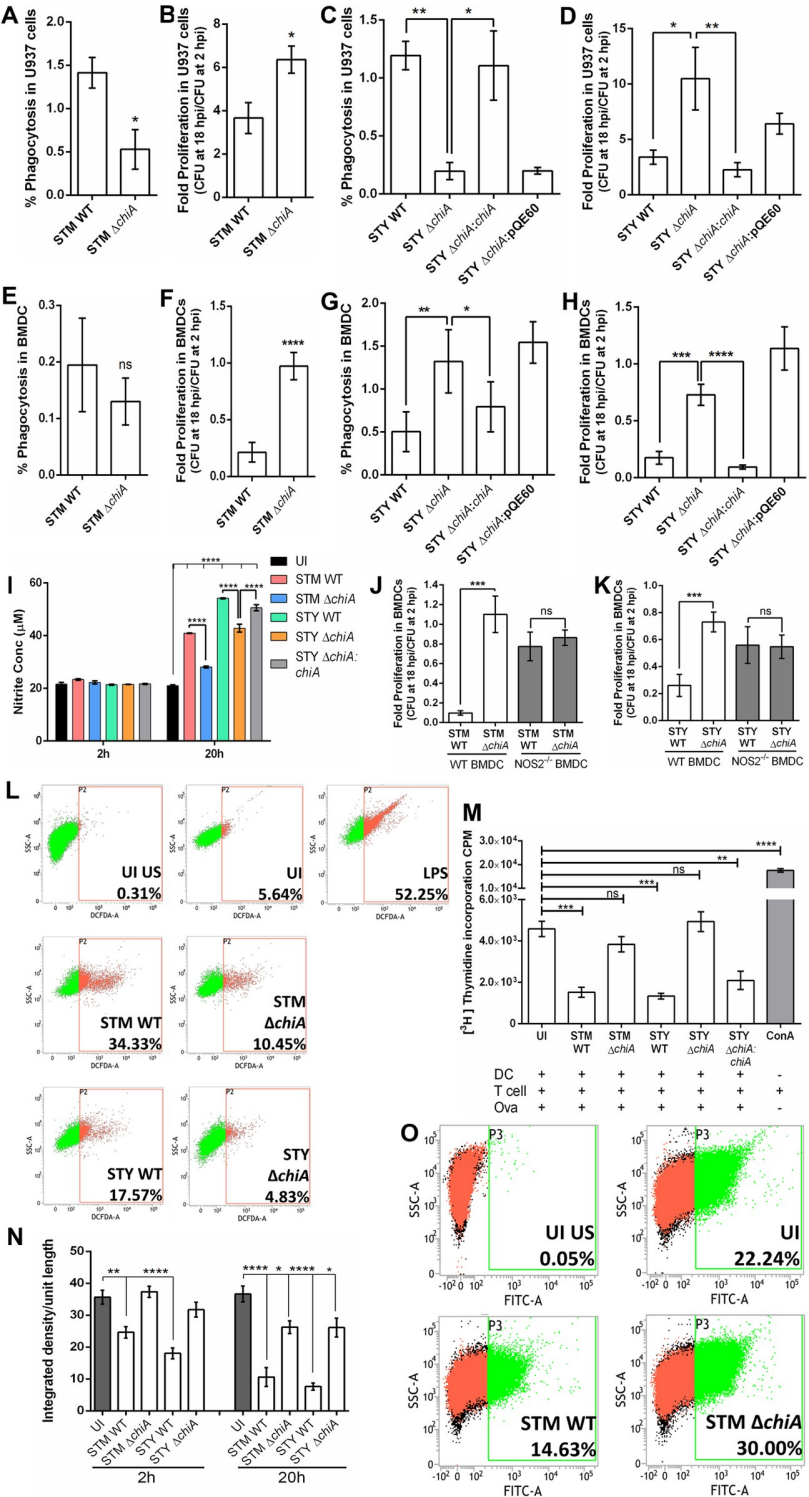
Chitinase induces NOS and ROS generation in phagocytic cells and inhibits antigen presentation. **(A)** % invasion and **(B)** fold proliferation of STM WT and STM Δ*chiA* strains, and **(C)** % invasion and **(D)** fold proliferation of STY WT, STY Δ*chiA*, STY Δ*chiA*:*chiA* and STY Δ*chiA*:pQE60 strains in U937 derived monocytes by gentamicin protection assay. (N = 3, n = 3). **(E)** % invasion and **(F)** fold proliferation of STM WT and STM Δ*chiA* strains, and **(G)** % invasion and **(H)** fold proliferation of STY WT, STY Δ*chiA*, STY Δ*chiA*:*chiA* and STY Δ*chiA*:pQE60 strains in BMDCs by gentamicin protection assay. (N = 3, n = 3). **(I)** Extracellular NO was estimated by Greiss assay from spent media obtained from STM WT, STM Δ*chiA*, STY WT, STY Δ*chiA* and STY Δ*chiA*:*chiA* infected BMDCs. (UI- Uninfected). (N = 3, n = 3). Two-way ANOVA was used to analyze the data. Intracellular survival of **(J)** STM WT and STM Δ*chiA* and **(K)** STY WT and STY Δ*chiA* strains was calculated in WT (NOS2^+/+^) and NOS2^-/-^ BMDCs by gentamicin protection assay. (N = 3, n = 3). **(L)** Representative flow cytometry plot for ROS estimation by DCFDA assay from STM WT, STM Δ*chiA*, STY WT, STY Δ*chiA* and STY Δ*chiA*:*chiA* infected and lipopolysaccharide (LPS; control) treated PMs. (UI US- Unstained and uninfected, UI- Uninfected). **(M)** ^3^H thymidine incorporation assay to assess CD8^+^ T cell proliferation 20 hpi with STM WT, STM Δ*chiA*, STY WT, STY Δ*chiA* and STY Δ*chiA*:*chiA* (UI- Uninfected, OVA- Ovalbumin, ConA- Concanavalin A). (N = 3, n = 6). **(N)** Quantification of the MHC-II density per unit length of the cell membrane of STM WT, STM Δ*chiA*, STY WT, STY Δ*chiA* and STY Δ*chiA*:*chiA* infected PMs after the indicated time (UI- Uninfected). (N = 2). **(O)** Representative flow cytometry plot showing surface MHC-II level on PMs infected with STM WT and STM Δ*chiA* for 20 hours (UI US- Unstained and uninfected, UI- Uninfected). Unpaired Student’s t test was used to analyze the data for **A, B, E, F, J, K** and one-way ANOVA was used to analyze the data for **C, D, G, H, M**.

After establishing a successful niche in the epithelial cells, Salmonella transcytoses to the lamina propria (LP) and infects the LP-resident macrophages and dendritic cells [28]. To understand the role of chitinase in phagocytic cell infection, we infected U937 monocytes and bone-marrow derived dendritic cells (BMDCs). Although the Δ*chiA* mutants were phagocyted less by U937 monocytes, they showed enhanced survival compared to the WT strains (**Fig 4A–4D** and **S2A and S2B**). While STM WT and STM Δ*chiA* were phagocytosed equally, STY Δ*chiA* showed increased phagocytosis and better survival in BMDCs than STY WT (**Figs 4E–4H** and **S2C and S2D**). We detected significantly less nitric oxide (NO) in the spent media from the Δ*chiA* mutant infected BMDCs (**Fig 4I**). Interestingly, both WT and Δ*chiA* mutant bacteria survived equally in NOS2^-/-^ BMDCs (**Fig 4J and 4K**). Furthermore, Δ*chiA* infected peritoneal macrophages (PM) showed significantly less ROS level than the WT bacteria-infected cells (**Fig 4L**), indicating that chitinase might be regulating RNI and ROS levels in the infected cells. To check the effect of NO on antigen presentation and T cell expansion, we quantified CD8^+^ T cell proliferation using OT1 transgenic mouse (C57BL/6-Tg(TcraTcrb) 1100Mjb/J). The TCR of this transgenic mouse recognizes OVA_257-264_ when presented by MHC-I molecules. This TCR recognition of MHC-I bound cognate peptide results in CD8^+^ T cell proliferation that can be measured by incorporation of ^3^H thymidine in the DNA of the proliferating population. We found that Δ*chiA* mutant infected BMDCs significantly expanded CD8^+^ T cells in response to the antigen stimulation (**Fig 4M**). Since macrophages and DCs possess MHC-I and MHC-II on the cell surface to induce CD8^+^ and CD4^+^ T cells population, respectively, we detected the surface MHC-II molecules on activated PMs. We found a significant reduction in the surface MHC-II level with WT infection, but not in Δ*chiA* infection (**Figs 4N and 4O and S2E and S2F**), indicating that *Salmonella* ChiA facilitates pathogen survival by dampening host antimicrobial responses.

### Chitinase facilitates *in vivo* invasion, survival, and pathogenesis of *Salmonella* Typhimurium

**Fig 5:**
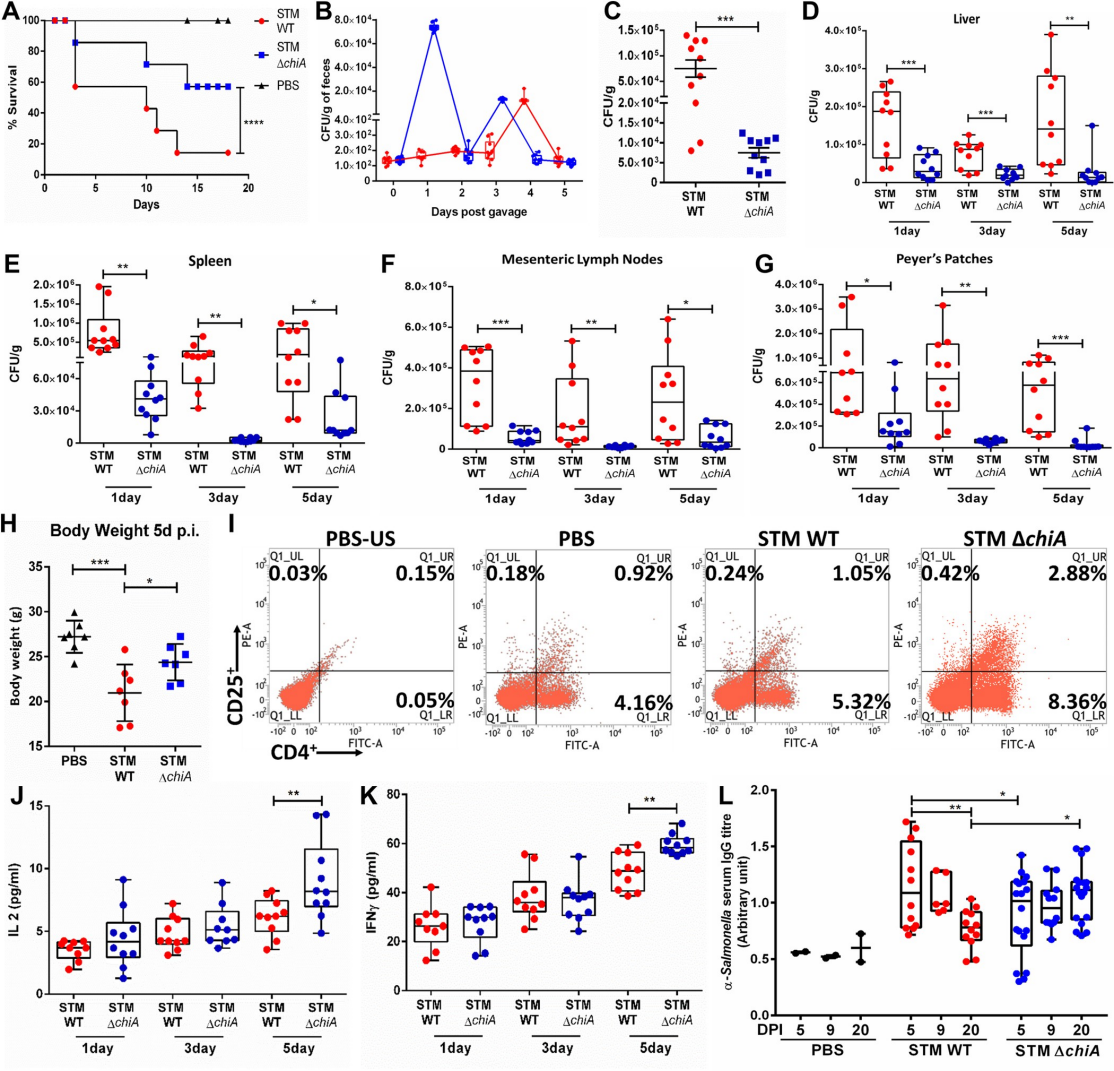
Chitinase facilitates *in vivo* invasion, survival, and pathogenesis of *Salmonella* Typhimurium. **(A)** Survival of the mice infected with a lethal dose of STM WT and STM Δ*chiA* (PBS = Phosphate Buffered Saline). Data are presented from one independent experiment, representative of 3 independent experiments (N = 3). **(B)** Bacterial shedding in the feces of STM WT and STM Δ*chiA* infected animals. Data are presented as mean ± SD of 3 independent experiment; each dot represents an individual animal (N = 3, n = 10). **(C)** *In vivo* invasion in PP by STM WT and STM Δ*chiA*. (N = 3). Bacterial burden in **(D)** liver, **(E)** spleen, **(F)** MLN and **(G)** PP of the infected mice after the indicated time with a sub-lethal dose of STM WT and STM Δ*chiA*. (N = 3). Unpaired Student’s t test was used to analyze the data for **C-G**. **(H)** Body weight of the infected mice 5 dpi with a sub-lethal dose of STM WT and STM Δ*chiA*. (N = 3). (PBS- Phosphate Buffered Saline). **(I)** Flow cytometry analysis of CD4^+^ and CD25^+^ T cells from total splenocytes isolated from STM WT and STM Δ*chiA* infected mice 20 dpi (US-PBS- Unstained splenocytes from PBS treated mouse). Data are presented from one independent experiment, representative of 3 independent experiments (N = 3). Pro-inflammatory cytokines **(J)** IL-2 and **(K)** IFN-γ level in serum from STM WT and STM Δ*chiA* infected mice after the indicated time. (N = 3); One-way ANOVA was used to analyze the data. **(L)** Serum anti-*Salmonella* antibody titer was quantified by sandwich ELISA after the indicated time. (N = 3); Two-way ANOVA was used to analyze the data.

To delineate the role of chitinase in *Salmonella* infection *in vivo*, we orally infected C57BL/6J mice with 10^8^ CFU of bacterial strains and monitored animal survival. The STM Δ*chiA* infected animals showed enhanced survival than the STM WT infected cohort (**Fig 5A**). We also found that STM Δ*chiA* bacteria were shed in the feces prior to the STM WT and the Δ*chiA* mutant was defective in Peyer’s patches (PP) colonization at 2 hpi (**Fig 5B and 5C**). Further, we orally infected C57BL/6J mice with a sublethal dose of *Salmonella* strains (10^7^ CFU/animal) and bacterial CFU from the liver, spleen, mesenteric lymph node (MLN), and PP were enumerated. We found that STM Δ*chiA* mutant infected animals showed less bacterial burden in each organ and bodyweight reduction than the STM WT infected animals (**Fig 5D–5H**). Additionally, STM Δ*chiA* infected mice showed a significantly reduced burden till 20 days post-infection (dpi; **S3A and S3B Fig**). We also found significantly enlarged spleens in STM Δ*chiA* infected mice 20 dpi (**S3C and S3D Fig**). We previously showed that STM Δ*chiA* infected spleens harbored fewer bacteria (**Fig 5E**); therefore, we hypothesized that STM Δ*chiA* infection leads to T cell activation and enlargement of the spleens. To validate this hypothesis and our *ex vivo* data showing the correlation between reduced NO induction and higher T cell activation by Δ*chiA* mutant, we isolated total splenocytes from the spleens of the STM Δ*chiA* infected animals and quantified the CD4^+^CD25^+^ T cell population by flow cytometry. Interestingly, we found that STM Δ*chiA* infection leads to a significant increase in the CD4^+^ and CD25^+^ T cells, as well as the double-positive CD4^+^CD25^+^ T cell population (**Fig 5I**). Analysis of T cell-mediated cytokine response revealed a significant increase in the pro-inflammatory cytokines IL-2 and IFN-γ in the serum isolated from Δ*chiA* infected animals (**Fig 5J and 5K**), while no difference was observed in the anti-inflammatory cytokine levels (**S3E and S3F Fig**). Previous reports suggested that high IFN-γ can induce B cell proliferation and enhance IgG2a and IgG3 production [29]. Therefore, we quantified the anti-*Salmonella* IgG titer from infected mice serum. Interestingly, we found a significant increase in the anti-*Salmonella* IgG titer in the serum obtained from STM Δ*chiA* infected cohort (**Fig 5L**). We further used the polyclonal convalescent sera from STM Δ*chiA* infected mice to probe against STM WT-mCherry whole cell lysate to test the reactivity of the sera. Multiple dense bands against various *Salmonella* proteins were obtained after incubating the membrane with sera collected from STM Δ*chiA* mutant infected cohort (**S3G Fig**). Together these data suggest that *Salmonella* chitinase A is essential for restricting innate and humoral immune responses i*n vivo*.

### Chitinase facilitates *Salmonella* Typhi colonization in *C*. *elegans*

**Fig 6:**
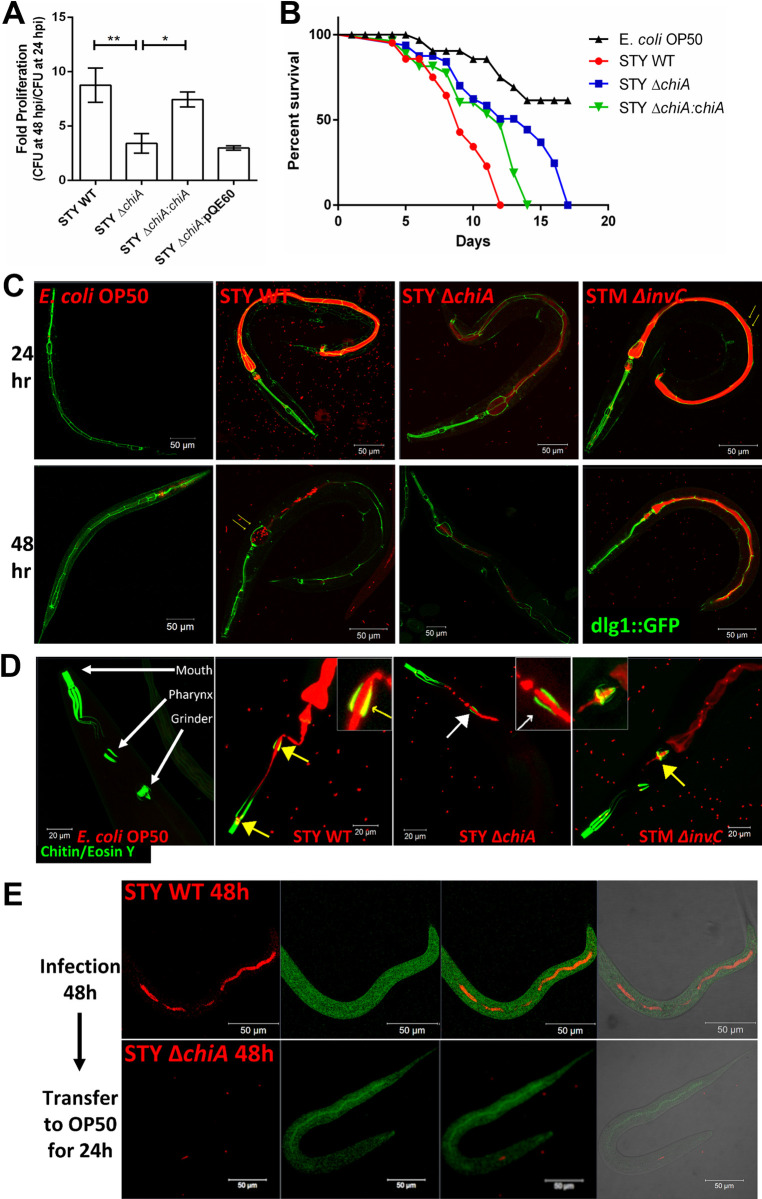
Chitinase enhances *Salmonella* Typhi virulence in *C*. *elegans*. **(A)** Bacterial fold change as the ratio of CFU obtained from the worms after 24 hours and 48 hours continuous feeding on STY WT, STY Δ*chiA*, STY Δ*chiA*:*chiA* and STY Δ*chiA*:pQE60 strains. (N = 4); One way ANOVA was used to analyze the data. **(B)** Survival of the worms fed on *E*. *coli* OP50, STY WT, STY Δ*chiA* and STY Δ*chiA*:*chiA*. Data are presented from one independent experiment, representative of 4 independent experiments (N = 4). **(C)** Representative images of bacterial colonization in worms gut as observed by infecting transgenic FT63 worms with mCherry expressing bacteria for the indicated time. Yellow arrows show the presence of intact bacteria in the terminal bulb of the worm. **(D)** Representative images of bacterial colonization on the chitin-rich organs of the worms as detected by eosin Y staining. Yellow arrows show colocalization of the bacteria (red) and the eosin-stained chitin-containing regions (green). White arrow shows the absence of colocalization of the bacteria and chitin-rich organs. **(E)** Representative images showing bacterial colonization and persistence in the worms’ gut.

**Fig 7:**
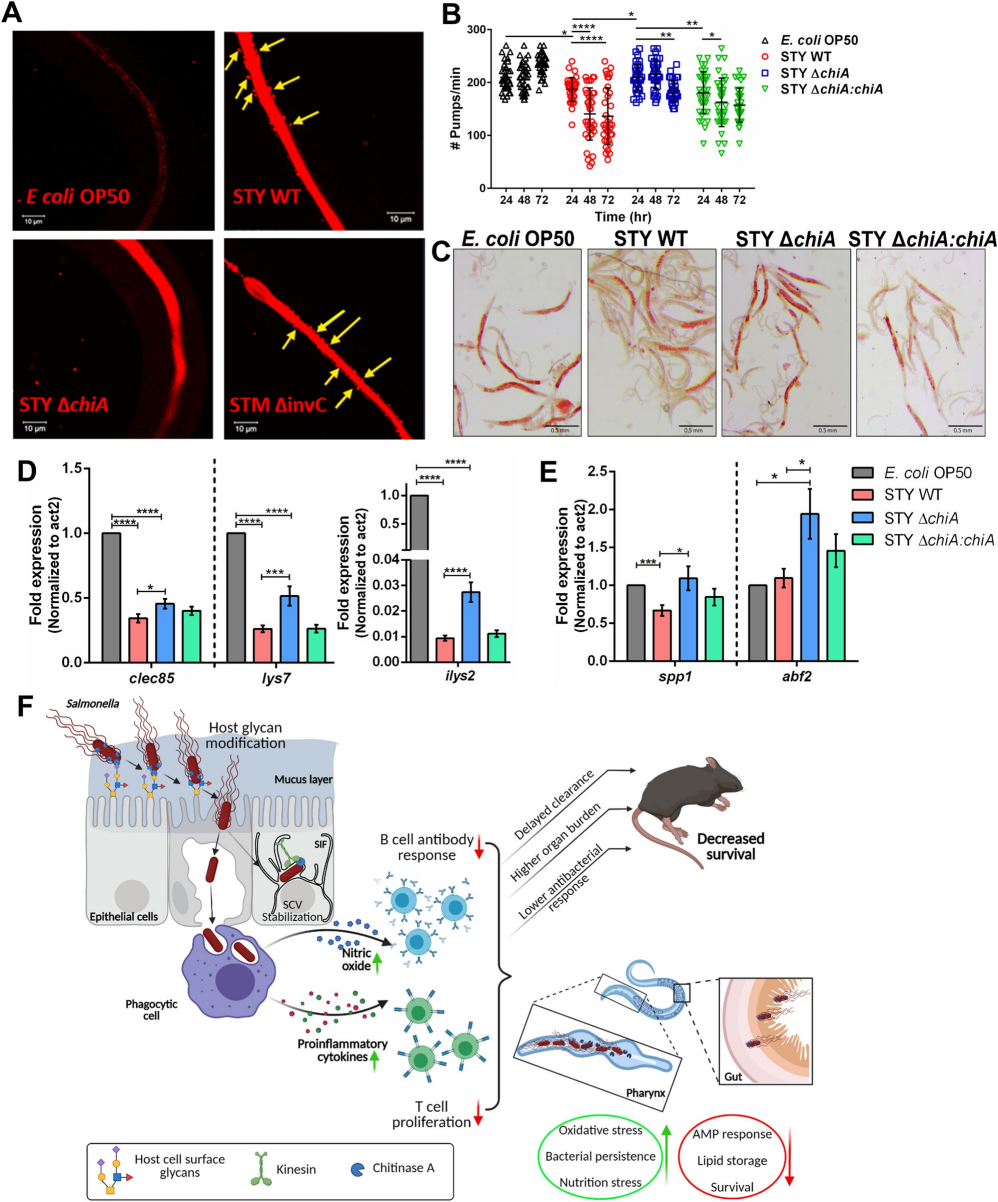
*Salmonella* chitinase is important for alteration of metabolism and antibacterial defense in *C*. *elegans*. **(A)** Representative images of bacterial colonization of the worms’ gut at higher magnification. Yellow arrows show the presence of the STY WT and STM Δ*invC* bacteria outside the gut lumen. **(B)** Quantification of no. of pharyngeal pumps/min of the worms at the indicated time. Data are represented as mean ± SD of 3 independent experiments (N = 3); Two-way ANOVA was used to analyze the data. **(C)** Representative images of Oil Red O (ORO) stained worms fed with different bacterial strains for 48 hours. **(D, E)** Quantitative RTPCR analysis of the p38 MAPK dependent antimicrobial peptide genes **(D)** *clec85*, *lys7*, *ilys2* and **(E)** *spp1* and *abf2* in worms fed with different bacterial strains for 48 hours. Fold change was normalized over *act2*. (N = 4). One-way ANOVA was used to analyze the data. **(F)** Model depicting the role of Chitinase A in bacterial invasion and regulation of host immune response during *Salmonella* pathogenesis in mouse and *C*. *elegans* host (created with Biorender.com).

Although *Salmonella* Typhi is an obligatory human pathogen that does not cause a significant infection in mice because of the presence of TLR11, however use of *Tlr11*^-/-^ mouse model has been reported to be largely inconsistent [30,31]. Long before the *Tlr11*^-/-^ mice model came into existence, Labrousse *et al*. suggested *Caenorhabditis elegans* could be used as an alternative host to study *S*. Typhi pathogenesis [32]. Given that *C*. *elegans* pharyngeal lumen is rich in chitin and chitinase substrate molecules, it served as a suitable host to study the role of chitinase in bacterial pathogenesis [33]. We checked the bacterial CFU in the infected worms after 24 hours (24hpi) and 48 hours (48hpi) of continuous feeding and found that the STY Δ*chiA* and STY Δ*chiA*:pQE60 strains showed a higher bacterial burden at 24hpi (**S3H Fig**), but the fold proliferation (CFU at 48hpi/CFU at 24hpi) of STY Δ*chiA* was lesser than that of the STY WT and STY Δ*chiA*:*chiA* strains (**Fig 6A**). Although these *Salmonella* Typhi strains were lethal to the nematodes, STY Δ*chiA* infected worms showed slower death (TD_50_ 330±8hrs) as compared to the STY WT (TD_50_ 190±10hrs) and STY Δ*chiA*:*chiA* (TD_50_ 270±12hrs) strains (**Fig 6B**). Together these data suggest that chitinase deletion reduces the virulence of *Salmonella* Typhi in *C*. *elegans*. We further checked bacterial colonization in the worm’s gut using the transgenic worm FT63 strain that expresses GFP in the epithelial cells. *S*. Typhi Δ*chiA* showed less colonization than STY WT at 24hpi, while the colonization was significantly reduced at 48hpi (**Fig 6C**). Several human pathogens such as *Salmonella* sp. and *Pseudomonas aeruginosa* have been reported to colonize the nematode gut lumen and cause gut distension [34]. Percent colonization as an indicator of gut distension was measured as the ratio of the diameter of the lumen occupied by the bacteria to the total diameter of the gut (**S3I Fig**). We next checked if *S*. Typhi utilizes chitinase to colonize the chitin-rich pharyngeal lumen by infecting N2 wildtype worms with different strains of *Salmonella* and stained the chitin-rich parts of the worms using eosin Y. Interestingly, after 24 hours of infection, luminal STY WT and STM Δ*invC* bacteria, but not STY Δ*chiA*, colocalized with the chitin-rich regions of the pharyngeal wall and terminal bulb (grinder; **Fig 6D**), indicating that *Salmonella* Typhi utilizes chitinase to colonize the chitin-rich pharynx and terminal bulb. Additionally, after 24 hours of feeding on STY Δ*chiA* followed by 24 hours feeding on *E*. *coli* OP50, the STY Δ*chiA* was unable to persist in the gut, whereas STY WT showed significantly higher colonization in the pharyngeal lumen (**S3J Fig**). Extended infection for 48 hours, followed by 24 hours of *E*. *coli* OP50 feeding revealed that STY WT could profoundly colonize the gut lumen, while STY Δ*chiA* colonization was diminished (**Fig 6E**). Interestingly, after 24 hours of continuous feeding, STY WT and STM Δ*invC* were found attached to the luminal wall leading to extra-intestinal tissue invasion, but not STY Δ*chiA*. (**Figs 7A and S4A and S4B**), suggesting that chitinase might be required to invade extra-intestinal tissues of the worms. To the best of our knowledge, this study is the first report suggesting an extra-intestinal invasion/colonization by *Salmonella* Typhi in *C*. *elegans*.

### *Salmonella* chitinase is important for alteration of metabolism and antibacterial defense in *C*. *elegans*

Since we observed that *Salmonella* uses chitinase to colonize chitin-rich organs (**Fig 6D**), such as the terminal bulb, the essential structure that breaks down bacterial cells to provide nutrition to the worms, we next looked into the nutritional state of the worms by counting the number of pharyngeal pumps per min. We found a gradual yet profound reduction in the number of pharyngeal pumps/min after 72 hours of STY WT and STY Δ*chiA*:*chiA* infection, beginning as early as 24hpi, while STY Δ*chiA* infected worms did not show pumping defect until 72hpi (**Fig 7B**). *In vivo* oxidative stress due to pathogen infection was quantified using CL2166 worms, that possess oxidative stress-inducible GFP. STY WT and STY Δ*chiA*:*chiA* infected worms showed significantly higher oxidative stress and ‘bag of worms’ phenotype (**S4C** and **S4D Fig**). Furthermore, we observed significantly less fat storage in worms infected with STY WT and STY Δ*chiA*:*chiA* in comparison to STY Δ*chiA* strain (**Figs 7C** and **S4E**). It has been reported that oxidative stress (high ROS), nutritional stress, and pathogen attack can induce the MAPK pathway in the worms, leading to apoptosis [35,36]. Therefore, we checked the level of phosphor-p38 and the RNA expression of several effector genes that are downstream of the MAPK pathway. Although phosphor-p38 MAPK (PMK-1) was upregulated in all three STY strains infected worms, equal transcriptional downregulation of *pmk1* and *mek1* was observed (**S4F and S4G Fig**) and PMK-1 regulated antimicrobial peptides were differentially expressed. While the expression of MAPK regulated antimicrobial peptide genes *clec85*, *lys7*, *ilys2* was downregulated in STY WT, STY Δ*chiA* and STY Δ*chiA*:*chiA* infected worms, their expression was partially rescued in the worms infected with STY Δ*chiA* bacteria (**Fig 7D**). Interestingly, STY Δ*chiA* infection completely rescued *spp1* expression and significantly upregulated *abf2* expression (**Fig 7E**), indicating an important function of chitinase in restricting the antimicrobial responses of the host.

## Discussion

*Salmonella* is a facultative intracellular human pathogen that has co-evolved with its host and has also developed various strategies to evade the host’s immune responses. Although *Salmonella* pathogenesis is governed by classical virulence factors such as adhesins, invasins, and toxins, emerging reports suggest that various unique metabolic proteins are important in various aspects of *Salmonella* pathogenesis. Several reports suggest that *Salmonella* can utilize a large pool of chemically diverse host nutrients, such as carbohydrates, lipids, amino acids, etc. [37]. Bacterial chitinases belong to GH18 and GH19, which are getting recognized as bacterial virulence factors along with several other structurally similar glycosidases such as sialidases, muraminidases, N-acetylgalactosaminidases, etc. [2]. Although *Salmonella chiA* was upregulated during infection, the role of this chitinase in *Salmonella* pathogenesis remains elusive. To answer this question, we generated isogenic Δ*chiA* mutant by the one-step gene inactivation method. Interestingly, we found that the mutant was invasion defective in epithelial cells. *Salmonella* injects several *Salmonella* pathogenicity island-1 (SPI1) effectors to induce bacterial entry into the epithelial cells [38]. Interestingly, the expression of two major SPI-1 encoded genes *invF* and *hilA* was significantly higher in the STM Δ*chiA* mutant strain, indicating that the bacteria overexpress these effectors to counter the lack of ChiA. Previous reports suggested that *Salmonella* remodels the host cell surface glycans to facilitate invasion in the epithelial cells [17–19]. Our observations from the lectin-binding assay indicate that chitinase aids in glycan remodeling by cleaving the terminal glycosyl molecules and making the mannose residues accessible to the bacteria for binding. We also found that Δ*chiA* mutants remain encased in SCVs as seen by EEA1-SCV colocalization at 15 mpi in Caco-2 cells until 10 hpi as seen by LAMP1-SCV colocalization, and after which the mutants escape the SCV and hyper-proliferate in the host cell cytoplasm. One conceivable explanation for this phenomenon could be limited nutrient availability. *Salmonella* utilizes an extended network of tubular vacuolar structures, known as *Salmonella*-induced filaments (SIFs), to acquire nutrients from the host cell cytoplasm [39]. SifA, a component of SIFs, interacts with SifA-Kinesin interacting protein (SKIP) to facilitate the recruitment of the motor protein kinesin-1 on the SCVs [40]. Interestingly, kinesin-1 and several other kinesin-like proteins (KIF18A, KIF17b, etc.) are heavily glycosylated, phosphorylated, and sumoylated post-translationally, and often the N-acetylglucosamine (GlcNAc) residues block the phosphorylation sites leading to disruption of the mobility of these motor proteins [41]. It is conceivable that chitinase being a glycoside hydrolase, could remove the GlcNAc residue to facilitate phosphorylation and mobility of the motor proteins. Therefore, in the strain lacking ChiA, this mobility is affected, leading to nutritional stress and quitting of the vacuoles. Additionally, the Δ*chiA* mutants were protected from phagocytes-mediated bacterial killing since the mutant bacteria-infected phagocytes showed reduced oxidative burst. NO is an important cell signaling molecule, produced against many human pathogens, such as *Salmonella*, *Mycobacterium*, *Listeria*, etc. [42]. Previous studies suggested that a low level of NO enhances T cell survival [43], while very high [NO] inhibit T cell proliferation [44]. Previous literature suggests that mammals also possess several GH18 family enzymes. Among these, chitotriosidase, acidic mammalian chitinase (true chitinases), and BRP-39/YKL-40 (CHI3L1; a chitinase-like protein or CLP) may have chitin-like targets and can modulate host immune responses during infections, allergy, tissue injury, inflammation, and tumor. Furthermore, BRP-39 was found to activate DCs and T cells and induce Th2 inflammatory responses [45]. Interestingly, CHI3L1 neutralization *in vivo* reduced *Salmonella* Typhimurium load in the peripheral organs, indicating a definitive role of this CLP in immune modulation during *Salmonella* pathogenesis [46]. Additionally, Ma *et al*. showed that CHI3L1 is a potent stimulator of lymphocyte activation gene 3 protein (LAG3), which in turn inhibits T cell activation [47]. Chitinase being a member of the same enzyme class, we can theorize that similar activities could be performed by chitinase as well. We also found that ChiA was important for downregulating the MHC-I molecules on the dendritic cells, leading to the inhibition of CD8^+^ T cell proliferation and subsequent antigen presentation. In coherence with the available literature [44], the enhanced T cell proliferation could be attributed to the absence of NO induction by the Δ*chiA* mutant strains. We further showed that the absence of *chiA* failed to downregulate the surface MHC-II molecules on the activated macrophages, which is a well-known phenomenon during *Salmonella* infection [48]. Existing literatures suggest that SPI-2 effector SteD stimulates E3 ubiquitin ligase MARCH8 mediated ubiquitination of MHCII, leading to its degradation and suppression of T cell-mediated adaptive immune responses [49,50]. Interestingly, MHCs also have complex glycosylation marks that often end with a terminal sialic acid residue [51]. The glycosylation status of MHC-I can regulate its structure, activity, stability, trafficking and spacing. Glc1Man9GlcNAc2 glycosylation on the MHC-I molecules facilitates its interaction with calnexin and calreticulin and regulates its folding and assembly, whereas improper interactions lead to MHC-I retention in the endoplasmic reticulum [52]. Therefore, further investigation is required to understand whether the glycoside hydrolase activity of chitinase could also contribute to the inhibition of endosomal recycling and MHC replenishment on the cell membrane. *In vivo* infection in C57BL/6 mice suggested that STM Δ*chiA* mutant could not invade the PP, leading to an early fecal shedding, a lower bacterial burden in different organs, enhanced pathogen clearance and increased host survival. Additionally, the sustained innate activated IFNγ production could be attributed to iNOS-mediated signaling in Δ*chiA* mutant infected mice [53]. Bhat *et al*. suggested that enhanced IFNγ production by cytotoxic CD8^+^ T cells can facilitate T cell mobility, proliferation, and cytolytic function during viral infection and cancer [54]. Analysis of total splenic lymphocytes by flow cytometry suggested that the Δ*chiA* mutant infected mice had an increased activated T cell population (CD4^+^CD25^+^) in the spleens, suggesting an intensified immune response in these mice. However, this needs to be explored further since *Salmonella* is known to induce immune tolerance in the chicken intestine by upregulating CD4^+^CD25^+^ regulatory T cells [55]. Activation of the adaptive immune responses was corroborated by significant increment in the pro-inflammatory cytokines and anti-STM IgG antibody titer in the STM Δ*chiA* infected mice sera. Invertebrates also possess a chitinase substrate, LacdiNAc, as cell surface glycans [11,12]. By infecting *C*. *elegans* with STY strains, we further showed that chitinase aids in bacterial attachment to the pharyngeal lumen as well as colonization and persistence in the worms. In addition, our data suggest that *Salmonella* Typhi chitinase might be important for extra-intestinal tissue invasion in the worms. Although the nematodes lack phagocytes-like specialized immune cells, during pathogen infections, *C*. *elegans* produce ROS, often localized to the host-pathogen interface [56]. Oxidative stress caused by pathogen infection and nutrient starvation leads to the worm bagging, which is the internal egg hatching [57]. This phenomenon was described by Aballay *et al*. in the case of *Salmonella* Typhimurium infection [34]. Our data suggest that *Salmonella* infection induces oxidative stress, leading to *“bag of worms”* formation. The host also employs the ROS detox system which is transcriptionally regulated by SKN-1 and DAF-16. SKN-1 induction and its nuclear localization is regulated by the p38 MAPK signaling pathway, which is comprised of NSY-1 (MAPKKK; ASK-1 homolog), SEK-1 (MAPKK) and PMK-1 (MAPK; p38 homolog) [58,59]. Although phospho-p38 (PMK-1) was increased in STY infection, this further validates that ROS induction leads to p38 activation and apoptosis [36]. Furthermore, *Salmonella* was reported to induce programmed cell death in germline cells by the LPS-Tol1 axis [35]. p38 MAPK pathway and DAF-16 also regulate antimicrobial response in *C*. *elegans* as the major defense mechanism. Among these, lysozyme family (LYS), *Ascaris suum* antibacterial factor family (ABF), saposin-like proteins family (SPP), and C-type lectins family (CLEC) have been shown to play an important role in the induced immune responses to bacterial infection [60]. We found significantly higher expression of fat-responsive antimicrobial peptides genes *spp1* and *abf2* when the worms were infected with the STY Δ*chiA* strain. SPP1 and ABF2 are predominantly found in the intestine and on the grinder, respectively [61], both of which are the sites of *Salmonella* attachment as found in this study. Together these data indicate a potential role of chitinase in modulating the innate immune response in the worms.

In summary, our results reveal that the glycoside hydrolase ChiA plays a wide range of crucial, although not indispensable, functions during *Salmonella* pathogenesis. Although speculative at this stage, some of our findings can be attributed to the moonlighting activity of chitinase and require further investigation regarding the structural and biochemical properties of this protein. Collectively, we showed that *Salmonella* Chitinase regulates different aspects of pathogenesis, ranging from aiding in invasion in the epithelial cells, impairing the activity of professional antigen-presenting cells to as diverse as immune response regulation in various hosts (**Fig 7F**), and emerges as a novel virulence factor.

## Materials and methods

### Ethics statement

The animal experiments were carried out in accordance with the approved guidelines of the Institutional Animal Ethics Committee at Indian Institute of Science, Bangalore, India (Registration No: 48/1999/CPCSEA). The Committee for the Purpose of Control and Supervision of Experiments on Animals (CPCSEA) is a statutory Committee, which is established under Chapter 4, Section 15(1) of the Prevention of Cruelty to Animals Act 1960. All procedures involving the use of animals were performed according to Institutional Animal Ethics Committee (IAEC) approved protocol by CPCSEA. Ethical clearance approval number for the study is CAF/Ethics/670/2019.

### Bacterial strains

All *Salmonella* Typhimurium strains used in this study are listed below with their genetic description. *Salmonella enterica* serovar Typhimurium strain 14028S was used as the wildtype strain and the parental background for all the mutant strains used in this study, i.e. Δ*chiA* and Δ*invC*. All strains were grown and maintained in Lennox broth (LB; 0.5% NaCl, 1% casein enzyme hydrolysate and 0.5% yeast extract) at 37°C under shaking conditions (180 rpm). *Salmonella enterica* serovar Typhi strain CT18 was used as the wildtype strain, and the parental background for the mutant strain used in this study, i.e., Δ*chiA*. *S*. Typhi *chiA* was trans-complemented in pQE60 plasmid in 5’ NcoI-*chiA*-BamHI 3’ direction. This plasmid was transferred to STY Δ*chiA* strain to make complement strain. Complemented strain STY Δ*chiA*:*chiA* and empty vector strain STY Δ*chiA*:pQE60 strains were maintained on LBA supplemented with ampicillin (50 μg/ml). The mCherry expressing strains were cultured in Lennox broth with 50μg/ml Ampicillin at 37°C in shaking condition.

### Isolation and maintenance of primary cells and cell lines

Human colorectal adenocarcinoma cell line Caco-2 (ATCC HTB-37) was cultured in complete DMEM media (Lonza), whereas human monocyte cell line U937 (ATCC CRL-1593.2) was maintained in complete RPMI 1640 media (Lonza) with 100 μM β-mercaptoethanol and differentiated to macrophages using 20 ng/ml PMA for 24 hours prior to infection. Bone-marrow was isolated from either wildtype (NOS2^+/+^) C57BL/6J mice or NOS2^-/-^ C57BL/6J mice as described previously [48]. Briefly, the tibia and femur bones were carefully taken out, caps were removed and the marrow was flushed with RPMI 1640 media using a 26G needle. After making single cell suspension, red blood cells (RBCs) were lysed using RBC lysis buffer. Cells were pelleted and grown in complete RPMI 1640 media supplemented with 20 ng/ml mGM-CSF (Peprotech), antibiotics and 100 μM β-mercaptoethanol. After every 2 days, the media was replenished. Once approximately 65–70% of the cells were differentiated to dendritic cells (loosely adherent spheres), the cells were collected and used for further experiments. To obtain peritoneal macrophages (PMs), thioglycolate was injected in the peritoneal cavity of C57BL/6J mice. After 5 days these mice were sacrificed and ice cold PBS was injected in the peritoneum to collect the peritoneal exudate. Any residual erythrocytes were lysed using RBC lysis buffer and the cells were maintained in complete RPMI 1640 media for further experiments.

### Generation of deletion mutant

Δ*chiA* mutant strains were made using one-step deletion strategy as mentioned by Datsenko and Wanner [20]. Briefly, wildtype *Salmonella* (*S*. Typhimurium 14028S or *S*. Typhi CT18) bacteria transformed with a ‘lambda red recombinase’ expressing plasmid under arabinose inducible promoter (pKD46), was grown in LB with 50 μg/ml ampicillin and was induced with 10 mM L-arabinose at 30°C to an OD_600_ of 0.35–0.4. Electrocompetent cells were prepared by pelleting the bacterial cells and washing the pellet three times with ice cold, sterile MiliQ water and 10% glycerol, followed by resuspension in 50 μl of 10% glycerol. Kanamycin resistance cassette was amplified from pKD4 plasmid using primers containing upstream and downstream sequences of *S*. Typhimurium *chiA* gene (STM14_0022) and *S*. Typhi *chiA* gene (STY0018) fragment. 500 ng of this PCR product was purified and used for electroporation. Transformants were selected on LB agar containing kanamycin plates and were further confirmed with confirmatory primers, *chiA* specific RT primers and kanamycin resistance cassette internal primers.

### Infection and gentamicin protection assay

Epithelial Caco-2 cell line was infected with mid-log phase culture of bacteria grown in LB (OD_600_ 0.3), whereas phagocytic U937 derived monocytes and BMDCs were infected with overnight culture (OD_600_ 0.3). The multiplicity of infection (MOI) of 10 was used in each case. Bacterial attachment to host cells was enhanced by centrifuging at 600 rpm for 10 min. After 25 min of infection, cells were treated with gentamicin (100 μg/ml in complete media) for 1 hour to remove extracellular bacteria and then maintained with 25 μg/ml gentamicin for the remainder of the experiment. 0.1% Triton-X 100 (v/v in 1x PBS) was used to lyse the cells and the lysate was plated on *Salmonella-Shigella* (SS) agar for *S*. Typhimurium strains and Wilson Blair (WB) agar for *S*. Typhi strains. For invasion assay, cells were lysed after incubation in 100 μg/ml gentamicin treatment (i.e., 1 hour post infection) and percent invasion was calculated with respect to the pre-inoculum used for infection. For intracellular survival assay (ICSA), infected cells were lysed at 2 hours and 18 hours post infection. CFU at 18 hours was divided by CFU at 2 hours to obtain fold replication of the intracellular bacteria. For estimating the cytoplasmic bacterial population, chloroquine resistance assay was performed [62]. Briefly, Caco-2 cells were infected by different bacterial strains as mentioned previously. The infected cells were treated with 800 μM chloroquine 1 hour prior to cell lysis and absolute CFU was calculated by plating the cell lysate on selective media.

### Quantitative RT-PCR

Bacterial RNA was isolated from infected cells as described previously by Eriksson *et al*.[14]. Briefly, *Salmonella* infected cells were lysed at different time intervals on ice by incubating for 30 minutes with 0.1% SDS, 1% acidic phenol and 19% ethanol in sterile water. Eukaryotic cell debris was removed by centrifuging the cell lysate at 300g for 10 minutes, followed by pelleting bacterial cells at 5000 rpm for 5 minutes. At each timepoint, bacteria were recovered from a 6-well plate of infected Caco-2 and pooled to isolate RNA. *In vitro* grown bacterial RNA was obtained by growing bacteria at 37°C in DMEM medium, under 5% CO_2_, without shaking. The bacterial pellet was resuspended in TRIzol reagent (Takara) and stored at -80°C. Young adult hermaphrodites were infected with respective bacterial strains for 48 hr. Infected worms were harvested by washing the plates with M9 buffer and pelleting at 1000g for 1 min. The extracellular bacteria were removed by repeatedly washing the pellet 5–6 times. The worms pellet was resuspended in TRIzol reagent (Takara) and stored at -80°C. RNA was isolated by phase separation method using chloroform. cDNA was synthesized with reverse transcriptase (GCC Biotech). Quantitative PCR was carried out using SYBR Green Q-PCR kit (Takara). Relative expression with respect to control (16s rRNA gene for bacterial genes and *act2* for *C*. *elegans* genes) was plotted as fold change.

### Lectin binding assay for cell surface glycan modification

Human colorectal carcinoma cells Caco-2 were infected with different bacterial strains as mentioned before. For confocal imaging, cells were seeded on coverslips prior to infection. After infection for the specified time, the cells were fixed with 3.5% PFA for 20 min on ice. For flow cytometry, cells were washed with PBS and treated with 1x Trypsin-EDTA (TE) for 15 min, under 5% CO_2_ at 37°C. After the cells were dislodged from the wells, TE was removed, and the cells were incubated with 1 ml complete media for 20 min under 5% CO_2_ at 37°C for recovery. To avoid non-specific lectin binding the cells were treated with blocking buffer (PBS+2% FBS) at RT for 15 min. Specific lectins (50μg/ml lectin solution in blocking buffer for every 10^6^ cells) (Vector Laboratories; #FL-1301, #FL-1071, #FL-1001) were added to each samples and incubated for 30 min at RT, followed by washing with blocking buffer. Cells treated with only FITC dye (Merck; #46950) were used as controls.

### Flow cytometry, immunofluorescence and immunoblot

Cells were fixed with 3.5% PFA for 20 min on ice. All staining except for the surface markers (MHC-II, CD4 and CD25), were performed in the presence of permeabilizing agent, 0.01% saponin (Sigma) dissolved in 2.5% BSA containing PBS. Flow cytometry analysis was carried out using BD FACSVerse and BD FACSAria and data were analyzed using BD FACSDiva software. Immunofluorescence images were obtained using Zeiss LSM 710 and/or Zeiss LSM 880. The images were analyzed using ZEN Black 2012 platform. For analysis of activated T cell population (CD4^+^ CD25^+^) from infected mice spleen, splenocytes were isolated from mice that survived through 20 days of infection. Total splenocytes were fixed using 3.5% PFA on ice for 20 min, followed by incubation for 1 hour at RT with fluorophore conjugated antibody cocktail in dark. The cells were washed with 1x PBS and analyzed by flow cytometry. After 48 hours of infection with indicated strains, *C*. *elegans* were harvested and washed 5 times with ice-cold M9 buffer. The worms were resuspended in homogenization buffer (HB: 15 mM HEPES pH 7.6, 10 mM KCl, 1.5 mM MgCl2, 0.1 mM EDTA, 0.5 mM EGTA, 44 mM sucrose and 1:10 PI cocktail) and sonicated with 40% amplitude and 30 sec pulses, 4–5 times, on ice. Equal quantities of proteins were resolved onto a 12% SDS-PAGE gel and transferred to 0.45 μm PVDF membrane using Trans-Blot semi dry transfer cell (Bio-Rad). Immunoblotting was performed to quantify phospho-p38 MAPK using β-actin as loading control. Anti-human LAMP1 (DSHB; #H4A3) and Anti-human EEA1 (CST; #3288) antibody was used for immunofluorescence microscopy. Anti-mouse I-A/I-E (or MHC-II) (clone 2G9) FITC (BD Pharmingen; #553623) antibody was used for immunofluorescence microscopy and flow cytometry. Anti-mouse CD4 FITC (Invitrogen; #11-0041-85), Anti-mouse CD25 PE (Invitrogen; #12-0251-82) antibodies were used for flow cytometry. Anti-human phospho-p38 MAPK (CST; #9211), Anti β-actin HRP (Imgenex; #IMG-5142A) antibodies were used for immunoblotting.

### Nitric oxide estimation

Sodium nitrite (Sigma) standards of 100 μM, 50 μM, 25 μM, 12.5 μM, 6.25 μM and 3.13 μM were prepared by diluting 0.1 M stock in deionized distilled water. Conditioned media from infected cells were collected after indicated time intervals for estimation of nitrite by Greiss assay [63]. 1% sulphanilamide solution was made in 5% phosphoric acid. To 50 μl of the standards and the samples (in triplicates), 50 μl acidic sulphanilamide was added and incubated at RT, in dark for 10 min. After incubation, 50 μl of 0.1% NED (N-1-naphthylethylene diamine dihydrochloride) solution was added and incubated for 10 min in dark at RT. OD_520_ was measured within 30 min of appearance of purple/magenta colored product using TECAN Infinite Pro 200 microplate reader.

### ROS measurement

Intracellular ROS was detected by 2’, 7’-dichlorofluorescein diacetate (H_2_DCFDA; Sigma) staining. Cells were stained with 10μM DCFDA at 37°C in dark. After 30 min, cells were washed with ice cold PBS and harvested followed by flow cytometry analysis at 495/530 nm in BD FACSVerse.

### T cell proliferation assay

WT BMDCs were infected by incubating the bacteria with DCs for 90min, followed by removal of the bacteria and incubating the infected cells with 25μg/ml gentamicin. Total splenocytes were isolated from the spleen of C57BL/6-Tg (TcraTcrb) 1100Mjb/J mice by mechanical disruption. Erythrocytes were lysed by RBC lysis buffer (Sigma) and cells were maintained in complete RPMI-1640. Finally, non-adherent cells were collected and were used for mixed lymphocyte proliferation assay. The proliferation of the lymphocytes in response to antigen stimuli, was detected by incorporation of the ^3^H_1_ as measured by the scintillation counter.

### *In vivo* experiment

6 weeks old male C57BL/6J mice were used for all the *in vivo* mice experiments. All animal experiments were approved by the Institutional Animal Ethics Committee (CAF/Ethics/670/2019) and the National Animal Care Guidelines were strictly followed. 10^8^ CFUs of overnight grown STM WT and STM Δ*chiA* mutant bacteria were used for oral infection for animal survival assay. The control group was orally administered with sterile 1x PBS. Animals were observed for 20 days for survival and body weight was documented. For *in vivo* invasion, the animals were euthanized after 2 hours of gavage, and the bacterial CFUs in Peyer’s patches (PP) were estimated. To check the bacterial shedding, fecal pellets were collected aseptically from the infected cohorts after the indicated time. Homogenates were plated on SS agar plates and CFUs were counted. For estimating *in vivo* bacterial burden in different organs, a sublethal dose of 10^7^ CFUs of each bacterial strain was used and bacterial CFUs from liver, spleen. MLN and PP were enumerated after indicated time intervals. Spleens were isolated from the animals after 20 days and the length was measured.

### ELISA for serum cytokines and anti-*Salmonella* IgG

Blood collected from infected animals by cardiac puncture under aseptic conditions, was incubated at RT to facilitate coagulation. Serum was then isolated by centrifugation at 5000 rpm for 10 min at RT and stored at -20°C for further use. Estimation of serum level of different pro-inflammatory cytokines (IL-2 and IFNγ) and anti-inflammatory cytokines (IL10 and IL4) was performed according to the manufacturer’s instructions. Anti-*Salmonella* IgG titer was measured by sandwich ELISA as mentioned previously [64]. Briefly, wells were coated with *Salmonella* LPS (200 ng/well; Sigma) at 4°C overnight. Next day, LPS was removed, and the wells were washed with PBST (PBS+0.05% Tween 20), followed by blocking for 1 hour at RT with 5% FBS in PBS to avoid non-specific binding. After blocking, wells were washed with PBST. The serum samples, diluted in blocking buffer, were added to the wells in triplicates and incubated for 2 hours. Subsequently, wells were washed with PBST and anti-mouse IgG (HRP conjugate) was then added to the wells and incubated for 1 hour at RT. Tetramethylbenzidine (TMB; Sigma) was added and the plate was incubated in dark for 20–30 min. The reactions were stopped with 2 N H_2_SO_4_ and the absorbance was measured at 450 nm.

### *In vivo* colonization in *Caenorhabditis* elegans

*C*. *elegans* var. Bristol worms wildtype strain N2, FT63 [xnIs17; dlg-1::GFP + rol-6(su1006)], and CL2166 [dvls19 III; dvls (pAF15)gst-4p::GFP::NLS III] strains were maintained on NGM media at 20°C. L4 or Young adult N2 hermaphrodite worms were used for *in vivo* experiments. 10^7^ CFU of different bacterial strains were seeded on NGM plates and grown for 16 hours. Young adult N2 worms were fed at 20°C with the different bacterial strains for 24 hours or 48 hours to check bacterial colonization in the worms [65]. Bacterial CFU was enumerated by plating worms’ lysate from an equal number of infected worms on WB agar plates. Fold change was calculated as the ratio of CFU after 48 hours to CFU after 24 hours. For confocal analysis of the worm gut colonization, FT63 worms were used. mCherry expressing bacterial strains were used to visualize the gut colonization.

To check worms survivability, 10^7^ CFUs of overnight grown bacterial strains were seeded on 30 mm dishes containing Brain Heart Infusion (BHI) agar media. ~30–40 young adult worms were added at the center of each plate and survival was monitored [66]. Animals were transferred to fresh bacterial plates every day for first 5 days and then after every 5 days. The worms were scored as live or dead at regular intervals throughout the course of the assay. Worms were considered dead when they failed to respond to touch stimulus.

Chitin-rich organs were visualized using Eosin Y stain. After 24 hours of infection, worms were harvested and washed 5 times with M9 buffer, followed by washing the worms pellet with citrate phosphate buffer (0.2 M Na_2_HPO_4_, 0.1 M potassium citrate, pH 6.0). The worms were resuspended in 500 μL citrate-phosphate buffer and 15 μL of 5 mg/ml eosin Y (in 70% ethanol) was added. Tubes were incubated at RT, in dark for 10 minutes, followed by centrifugation at 1000g for 1 min for washing. The supernatant was discarded and the pellet was washed with citrate phosphate buffer 5 times to remove excess eosin Y.

The effect of bacterial colonization was determined by infecting CL2166 worms for 48 hours with different strains. CL2166 worms possess oxidative stress inducible GFP. Fluorescence of the infected worms was visualized using Zeiss LSM 880 with Multiphoton mode.

### Bacterial persistence assay in *C*. *elegans*

Young adult N2 worms were infected as mentioned previously. After 24 hours or 48 hours of infection, the worms were harvested and washed 5 times with M9 buffer. After indicated time, ~30 worms were mounted for confocal imaging. Rest of the worms were transferred to *E*. *coli* OP50 plate for further 24 hours. These worms were harvested, washed and imaged as mentioned previously.

### Quantification of pharyngeal pumps

The effect of bacterial colonization on the chitin-rich grinder integrity was determined by counting the number of pharyngeal pumps per min. Young adult worms were infected as mentioned previously. After indicated infection time, no. of pharyngeal pumps/min was counted for ~25 worms from each infected plate.

### Fat estimation by Oil red O

Neutral lipids present in the worms was estimated by Oil Red O (ORO; Sigma) staining [67]. Briefly, solution of Oil Red O was prepared in isopropanol (5 mg/ml) and diluted to 60% in water before use. Synchronized L4 animals were allowed to feed on *E*. *coli* and STY strains for 48 hr. Worms were harvested in M9 buffer, followed by fixing and permeabilizing using MRWB buffer (160 mM KCl, 40 mM NaCl, 14 mM Na_2_-EGTA, 1 mM spermidine-HCl, 0.4 mM spermine, 30 mM Na-PIPES [Na-piperazine N, N’-bis(2-ethanesulfonic acid); pH 7.4], 0.2% β-mercaptoethanol, 0.2% paraformaldehyde) for 1 hour at RT. The animals were stained with 60% ORO at RT. Excess strain was removed by washing twice with 1x PBST (PBS+0.01% Tween 20). Stained animals were mounted on agar pads.

### Statistical analysis

Data were plotted using GraphPad Prism 6 software. Statistical analysis was performed using Student’s t-test or ANOVA as indicated. The results are presented as mean ± SEM, unless mentioned otherwise. p values <0.05 was considered to be significant (p values: ****<0.0001, ***<0.001, **<0.01, *<0.05).

## Supporting information

S1 Fig *Salmonella* ChiA is non-essential for *in vitro* growth and *chiA* deletion interferes with the intracellular life of the pathogen. **(A)** BLAST analysis showing the identity of chitinase A of *Salmonella* serovars Typhimurium and Typhi with known pathogenic chitinases and chitin-binding proteins. Growth analysis of LB grown cultures of **(B)** STM WT and STM Δ*chiA*, **(C)** STY WT, STY Δ*chiA*, STY Δ*chiA*:*chiA* and STY Δ*chiA*:pQE60. Absolute CFU/ml values of **(D)** STM WT and STM Δ*chiA*, **(E)** STY WT, STY Δ*chiA*, STY Δ*chiA*:*chiA* and STY Δ*chiA*:pQE60 in Caco2 cells in gentamicin protection assay after the indicated time. Data are represented as mean ± SEM of 3 independent experiments (N = 3, n = 3). **(F)** Mean Fluorescence Intensity (MFI) of Neu5Ac-bound SNA-FITC, Gal-bound PNA-FITC and mannose-bound ConA-FITC lectins on Caco2 cells 30 mpi and 120 mpi with STM WT, STM Δ*chiA*, STY WT and STY Δ*chiA* (UI- Uninfected). Data are represented as mean ± SEM of 2 independent experiments (N = 2). Two-way ANOVA was used to analyze the data. % Colocalization of mCherry expressing (red) **(G)** STM WT and STM Δ*chiA*, **(H)** STY WT and STY Δ*chiA* with LAMP1 (green) in Caco-2 cells at 2/10/16 hpi. Data are represented as mean ± SEM of 3 independent experiments (N = 3). Unpaired Student’s t test was used to analyze the data**. (I)** % Colocalization of mCherry expressing (red) bacteria with EEA1 (green) in Caco-2 cells at 15/30/120 mpi. Data are represented as mean ± SD of 2 independent experiments (N = 2). One-way ANOVA was used to analyze the data.
(TIF)

S2 Fig *Salmonella* chitinase regulates host immune responses surface MHC on antigen-presenting cells. Absolute CFU/ml values of **(A)** STM WT and STM Δ*chiA*, **(B)** STY WT, STY Δ*chiA*, STY Δ*chiA*:*chiA* and STY Δ*chiA*:pQE60 in U937 derived monocytes in gentamicin protection assay after the indicated time. Data are represented as mean ± SEM of 3 independent experiments (N = 3, n = 3). Absolute CFU/ml values of **(C)** STM WT and STM Δ*chiA*, **(D)** STY WT, STY Δ*chiA*, STY Δ*chiA*:*chiA* and STY Δ*chiA*:pQE60 in murine BMDCs in gentamicin protection assay after the indicated time. Data are represented as mean ± SEM of 3 independent experiments (N = 3, n = 3). **(E)** Representative flow cytometry plot showing surface MHC-II level on PMs infected with STY WT, STY Δ*chiA* and STY Δ*chiA*:*chiA* for 20 hours (UI US- Unstained uninfected, UI- Uninfected, STY Δ*c*:*chiA*- STY Δ*chiA*:*chiA*). **(F)** Representative images showing surface MHC-II on PMs infected with STM WT, STM Δ*chiA*, STY WT and STY Δ*chiA* for the indicated time. PMs were stained for surface MHCII without any permeabilizing agent (UI- Uninfected).
(TIF)

S3 Fig *chiA* deletion impairs *in vivo* pathogenesis of *Salmonella*. Bacterial CFU in **(A)** spleen and **(B)** liver from STM WT and STM Δ*chiA* infected mice after the indicated time. **(C)** STM WT and STM Δ*chiA* infected mice spleen length were measured after 5 days and 20 days of oral infection. Data are presented from 3 independent experiments. One-way ANOVA was used to analyze the data. **(D)** Representative images of spleens isolated from STM WT and STM Δ*chiA* infected mice after 20 days. Data are presented from one independent experiment, representative of 3 independent experiments (N = 3). Anti-inflammatory cytokine **(E)** IL-10 and **(F)** IL-4 level in serum from STM WT and STM Δ*chiA* infected mice after the indicated time. Data are presented as mean ± SEM of 3 independent experiments (N = 3). One-way ANOVA was used to analyze the data. **(G)** Representative immunoblots showing the reactivity of the STM WT and STM Δ*chiA* infected mice sera against mCherry-tagged *Salmonella* whole cell lysate. Coomassie Brilliant Blue stained gel shows equal loading in all the lanes. Furthermore, the blot was probed with anti-mCherry antibody. The Data are presented from 2 independent experiments (N = 2). **(H)** Quantification of absolute bacterial CFU obtained from infected *C*. *elegans* after 24 hours and 48 hours continuous feeding on STY WT, STY Δ*chiA*, STY Δ*chiA*:*chiA* and STY Δ*chiA*:pQE60 strains. Data are represented as mean ± SEM of 4 independent experiments. **(I)** % colonization of the worms gut after 24 hours of continuous feeding with different bacterial strains. Data are represented as mean ± SEM of 4 independent experiments. One-way ANOVA was used to analyze the data. **(J)** Representative images showing bacterial colonization and persistence in the worms’ gut after shorter exposure.
(TIF)

S4 Fig Chitinase helps in extra-intestinal colonization and immune regulation in *C*. *elegans*. Representative images of parts of FT63 worms gut showing extra-intestinal invasion of the STY WT strain after 48 hours of continuous feeding **(A)** at higher magnification (scale bar- 5 μm) and **(B)** at lower magnification (scale bar- 10 μm). Yellow arrows show the presence of STY WT and STM Δ*invC* bacteria outside the gut lumen (Green). **(C)** Representative images of CL2166 worms after 48 hours of feeding on STY strains. GFP fluorescence is indicative of oxidative stress. Insets show ‘bag of worms’ resulting from oxidative stress in the STM WT and STM Δ*chiA*:*chiA* infected worms. **(D)** Quantification of GFP MFI in CL2166 worms. Data are represented as mean ± SEM of 3 independent experiments. One-way ANOVA was used to analyze the data. **(E)** Quantification of the ORO-stained parts of the worms fed with different bacterial strains for 48 hours. Data are represented as mean ± SEM of 3 independent experiments. Two-way ANOVA was used to analyze the data. **(F)** Immunological detection of phospho-p38 MAPK (PMK-1) from worms fed with *E*. *coli* OP50 and STY strains for 48 hours. β-actin was used as loading control. **(G)** qRTPCR analysis of the p38 MAPK (PMK-1) pathway genes *pmk1* and *mek1* in worms fed with *E*. *coli* OP50 and STY strains for 48 hours. Fold change was normalized over *act2*. Data represent mean ± SEM of 4 independent experiments. One-way ANOVA was used to analyze the data.
(TIF)
