# Supplementary figures and images for: GH18 family glycoside hydrolase Chitinase A of Salmonella enhances virulence by facilitating invasion and modulating host immune responses
Source: PLoS Pathog. 2022 Apr 28;18(4):e1010407. doi: 10.1371/journal.ppat.1010407 (PMC9049553; doi:10.1371/journal.ppat.1010407)

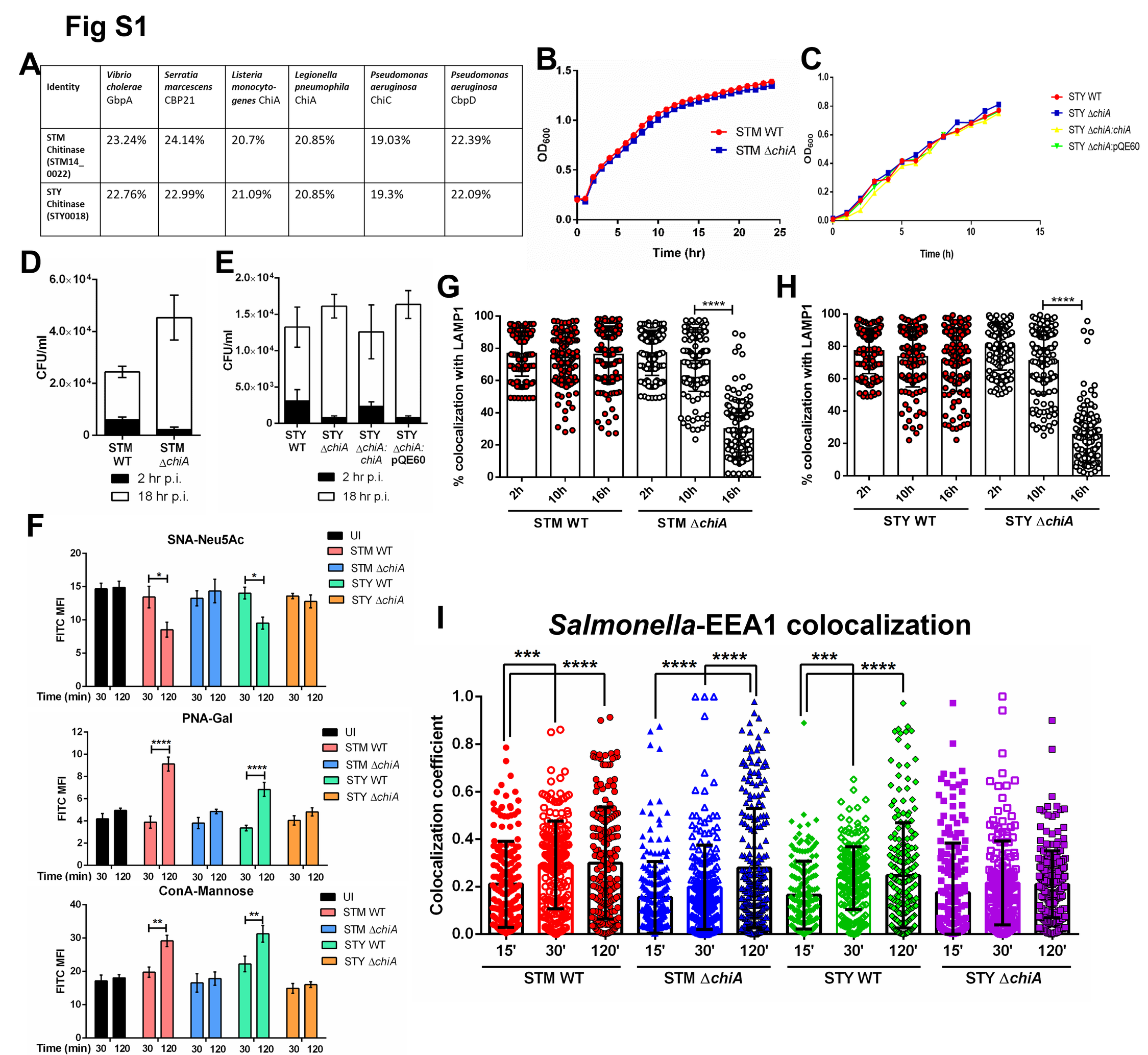

Supplement: S1 Fig — (A) BLAST analysis showing the identity of chitinase A of Salmonella serovars Typhimurium and Typhi with known pathogenic chitinases and chitin-binding proteins. Growth analysis of LB grown cultures of (B) STM WT and STM ΔchiA, (C) STY WT, STY ΔchiA, STY ΔchiA:chiA and STY ΔchiA:pQE60. Absolute CFU/ml values of (D) STM WT and STM ΔchiA, (E) STY WT, STY ΔchiA, STY ΔchiA:chiA and STY ΔchiA:pQE60 in Caco2 cells in gentamicin protection assay after the indicated time. Data are represented as mean ± SEM of 3 independent experiments (N = 3, n = 3). (F) Mean Fluorescence Intensity (MFI) of Neu5Ac-bound SNA-FITC, Gal-bound PNA-FITC and mannose-bound ConA-FITC lectins on Caco2 cells 30 mpi and 120 mpi with STM WT, STM ΔchiA, STY WT and STY ΔchiA (UI- Uninfected). Data are represented as mean ± SEM of 2 independent experiments (N = 2). Two-way ANOVA was used to analyze the data. % Colocalization of mCherry expressing (red) (G) STM WT and STM ΔchiA, (H) STY WT and STY ΔchiA with LAMP1 (green) in Caco-2 cells at 2/10/16 hpi. Data are represented as mean ± SEM of 3 independent experiments (N = 3). Unpaired Student’s t test was used to analyze the data. (I) % Colocalization of mCherry expressing (red) bacteria with EEA1 (green) in Caco-2 cells at 15/30/120 mpi. Data are represented as mean ± SD of 2 independent experiments (N = 2). One-way ANOVA was used to analyze the data. (TIF) [file ppat.1010407.s001.tif]

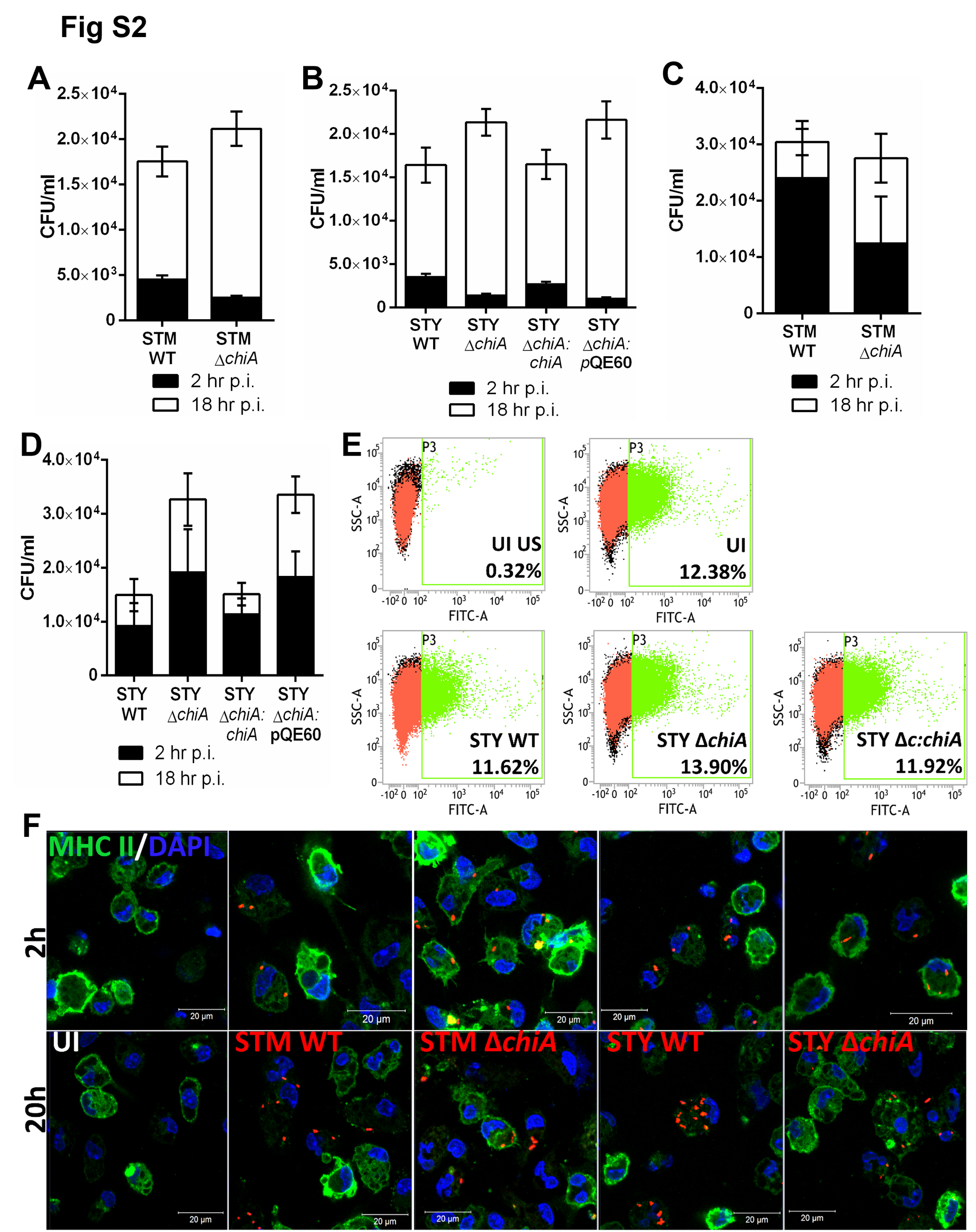

Supplement: S2 Fig — Absolute CFU/ml values of (A) STM WT and STM ΔchiA, (B) STY WT, STY ΔchiA, STY ΔchiA:chiA and STY ΔchiA:pQE60 in U937 derived monocytes in gentamicin protection assay after the indicated time. Data are represented as mean ± SEM of 3 independent experiments (N = 3, n = 3). Absolute CFU/ml values of (C) STM WT and STM ΔchiA, (D) STY WT, STY ΔchiA, STY ΔchiA:chiA and STY ΔchiA:pQE60 in murine BMDCs in gentamicin protection assay after the indicated time. Data are represented as mean ± SEM of 3 independent experiments (N = 3, n = 3). (E) Representative flow cytometry plot showing surface MHC-II level on PMs infected with STY WT, STY ΔchiA and STY ΔchiA:chiA for 20 hours (UI US- Unstained uninfected, UI- Uninfected, STY Δc:chiA- STY ΔchiA:chiA). (F) Representative images showing surface MHC-II on PMs infected with STM WT, STM ΔchiA, STY WT and STY ΔchiA for the indicated time. PMs were stained for surface MHCII without any permeabilizing agent (UI- Uninfected). (TIF) [file ppat.1010407.s002.tif]

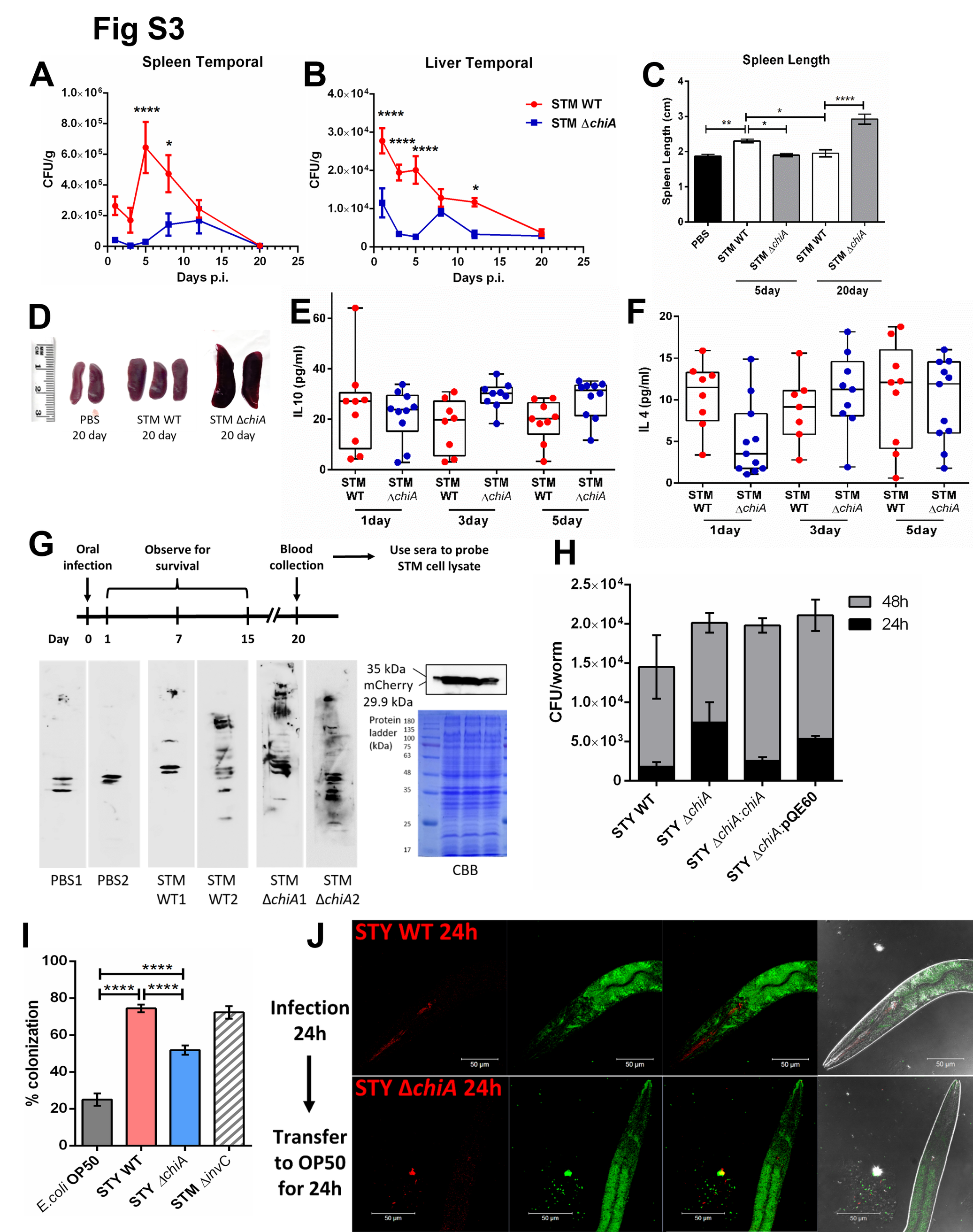

Supplement: S3 Fig — Bacterial CFU in (A) spleen and (B) liver from STM WT and STM ΔchiA infected mice after the indicated time. (C) STM WT and STM ΔchiA infected mice spleen length were measured after 5 days and 20 days of oral infection. Data are presented from 3 independent experiments. One-way ANOVA was used to analyze the data. (D) Representative images of spleens isolated from STM WT and STM ΔchiA infected mice after 20 days. Data are presented from one independent experiment, representative of 3 independent experiments (N = 3). Anti-inflammatory cytokine (E) IL-10 and (F) IL-4 level in serum from STM WT and STM ΔchiA infected mice after the indicated time. Data are presented as mean ± SEM of 3 independent experiments (N = 3). One-way ANOVA was used to analyze the data. (G) Representative immunoblots showing the reactivity of the STM WT and STM ΔchiA infected mice sera against mCherry-tagged Salmonella whole cell lysate. Coomassie Brilliant Blue stained gel shows equal loading in all the lanes. Furthermore, the blot was probed with anti-mCherry antibody. The Data are presented from 2 independent experiments (N = 2). (H) Quantification of absolute bacterial CFU obtained from infected C. elegans after 24 hours and 48 hours continuous feeding on STY WT, STY ΔchiA, STY ΔchiA:chiA and STY ΔchiA:pQE60 strains. Data are represented as mean ± SEM of 4 independent experiments. (I) % colonization of the worms gut after 24 hours of continuous feeding with different bacterial strains. Data are represented as mean ± SEM of 4 independent experiments. One-way ANOVA was used to analyze the data. (J) Representative images showing bacterial colonization and persistence in the worms’ gut after shorter exposure. (TIF) [file ppat.1010407.s003.tif]

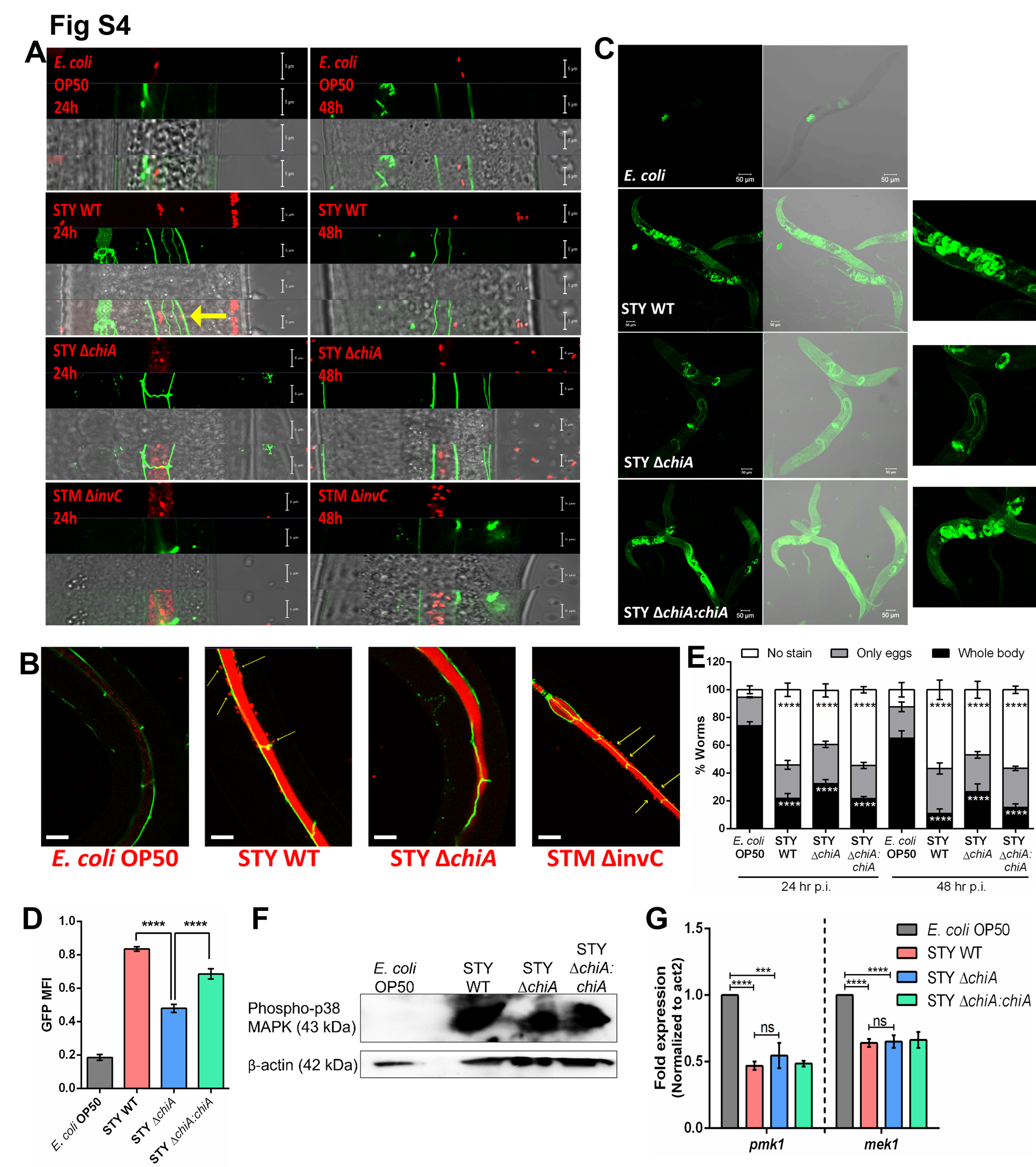

Supplement: S4 Fig — Representative images of parts of FT63 worms gut showing extra-intestinal invasion of the STY WT strain after 48 hours of continuous feeding (A) at higher magnification (scale bar- 5 μm) and (B) at lower magnification (scale bar- 10 μm). Yellow arrows show the presence of STY WT and STM ΔinvC bacteria outside the gut lumen (Green). (C) Representative images of CL2166 worms after 48 hours of feeding on STY strains. GFP fluorescence is indicative of oxidative stress. Insets show ‘bag of worms’ resulting from oxidative stress in the STM WT and STM ΔchiA:chiA infected worms. (D) Quantification of GFP MFI in CL2166 worms. Data are represented as mean ± SEM of 3 independent experiments. One-way ANOVA was used to analyze the data. (E) Quantification of the ORO-stained parts of the worms fed with different bacterial strains for 48 hours. Data are represented as mean ± SEM of 3 independent experiments. Two-way ANOVA was used to analyze the data. (F) Immunological detection of phospho-p38 MAPK (PMK-1) from worms fed with E. coli OP50 and STY strains for 48 hours. β-actin was used as loading control. (G) qRTPCR analysis of the p38 MAPK (PMK-1) pathway genes pmk1 and mek1 in worms fed with E. coli OP50 and STY strains for 48 hours. Fold change was normalized over act2. Data represent mean ± SEM of 4 independent experiments. One-way ANOVA was used to analyze the data. (TIF) [file ppat.1010407.s004.tif]
